# Deep geometric representations for modeling effects of mutations on protein-protein binding affinity

**DOI:** 10.1371/journal.pcbi.1009284

**Published:** 2021-08-04

**Authors:** Xianggen Liu, Yunan Luo, Pengyong Li, Sen Song, Jian Peng

**Affiliations:** 1 Laboratory for Brain and Intelligence and Department of Biomedical Engineering, Tsinghua University, Beijing, China; 2 School of Computing and Artificial Intelligence, Southwest Jiaotong University, Chengdu, China; 3 Department of Computer Science, University of Illinois at Urbana-Champaign, Urbana, Illinois, United States of America; 4 Beijing Innovation Center for Future Chip, Tsinghua University, Beijing, China; Fox Chase Cancer Center, UNITED STATES

## Abstract

Modeling the impact of amino acid mutations on protein-protein interaction plays a crucial role in protein engineering and drug design. In this study, we develop GeoPPI, a novel structure-based deep-learning framework to predict the change of binding affinity upon mutations. Based on the three-dimensional structure of a protein, GeoPPI first learns a geometric representation that encodes topology features of the protein structure via a self-supervised learning scheme. These representations are then used as features for training gradient-boosting trees to predict the changes of protein-protein binding affinity upon mutations. We find that GeoPPI is able to learn meaningful features that characterize interactions between atoms in protein structures. In addition, through extensive experiments, we show that GeoPPI achieves new state-of-the-art performance in predicting the binding affinity changes upon both single- and multi-point mutations on six benchmark datasets. Moreover, we show that GeoPPI can accurately estimate the difference of binding affinities between a few recently identified SARS-CoV-2 antibodies and the receptor-binding domain (RBD) of the S protein. These results demonstrate the potential of GeoPPI as a powerful and useful computational tool in protein design and engineering. Our code and datasets are available at: https://github.com/Liuxg16/GeoPPI.

## Author summary

Estimating the binding affinities of protein-protein interactions (PPIs) is crucial to understand protein function and design new functional proteins. Since the experimental measurement in wet-labs is labor-intensive and time-consuming, fast and accurate *in silico* approaches have received much attention. Although considerable efforts have been made in this direction, predicting the effects of mutations on the protein-protein binding affinity is still a challenging research problem. In this work, we introduce GeoPPI, a novel computational approach that uses deep geometric representations of protein complexes to predict the effects of mutations on the binding affinity. The geometric representations are first learned via a self-supervised learning scheme and then integrated with gradient-boosting trees to accomplish the prediction. We find that the learned representations encode meaningful patterns underlying the interactions between atoms in protein structures. Also, extensive tests on major benchmark datasets show that GeoPPI has made an important improvement over the existing methods in predicting the effects of mutations on the binding affinity.

## Introduction

Protein-protein interactions (PPIs) play an essential role in many fundamental biological processes. As a representative example, the antibody (Ab) is a central component of the human immune system that interacts with its target antigen to elicit an immune response. This interaction is performed between the complementary determining regions (CDRs) of the Ab and a specific epitope on the antigen. The antibody-antigen binding is specific and selective and has made antibody therapy widely used for a broad range of diseases including several types of cancer [1] and viral infection [2].

The binding affinity (also called the binding free energy), Δ*G*, is usually used to measure the thermodynamics of protein-protein interactions. Despite the broad application potentials of Ab therapy, it is very challenging to design Abs that have a desired binding affinity with antigens [3]. One of the solutions is to identify affinity-enhancing mutations based on Ab templates [4–6]. However, this strategy faces two-fold challenges when implemented in wet-labs. On the one hand, the experimental measurement of the mutation effects is a labor-intensive and time-consuming process. On the other hand, the space of possible Ab mutants is combinatorially large, for which existing methods cannot explore exhaustively in a reasonable timeframe. Therefore, fast and inexpensive *in silico* evaluation of binding affinity changes upon mutations (i.e., ΔΔ*G*) is a promising alternative for screening affinity-enhancing mutations in protein engineering and antibody design.

Traditional methods for computationally modeling the binding affinity changes upon mutations can be grouped into three categories: 1) the molecular modeling approach, which simulates the difference of free energy between two states of a system (i.e., the wild type and the mutant) based on continuum solvent models [7], 2) empirical energy-based methods, leveraging classical mechanics or statistical potentials to calculate the free-energy changes [8], and 3) machine learning based methods that fit the experimental data using sophisticated engineered features of the changes in structures. The methods in the first category, such as coarse-grained molecular dynamics simulations [9, 10], usually provide reliable simulation results but require heavy computational resources, which limits their applicability [11]. The empirical energy-based methods, exemplified by STATIUM [12], FoldX [8] and Discovery Studio [13], have accelerated the prediction but suffered from insufficient conformational sampling, especially for mutations in flexible regions.

The accumulating of experimental data has provided an unprecedented opportunity for machine learning methods to directly model the intrinsic relationship between a mutation and the resulting change on binding affinity. In particular, Geng et al. [14] used a limited number of predictive features derived from interface structure, evolution and approximated energy, as the input of a random forest model to predict the affinity changes upon mutations. Similarly, MutaBind2 [15] introduced seven features, including interactions of proteins with the solvent, evolutionary conservation, and thermodynamic stability of complexes, for the prediction of the affinity changes upon mutations, which achieved the state-of-the-art performance on the SKEMPI 2.0 dataset [16]. Most of the existing machine-learning methods use physical quantities as input features, which require considerable computational time to solve [17, 18]. In addition, these input features are mostly manually engineered based on the known rules in protein structures, often limiting their predictive generalization across various protein structures. In this work, we aim for developing a method that not only provides fast and accurate predictions, but also generalizes well on the unseen protein structures.

In this paper, we propose a novel framework, called GeoPPI, to accurately model the effects of mutations on the binding affinity based on geometric representations learned from protein structures. GeoPPI comprises two components, a geometric encoder and a gradient-boosting tree (GBT). The geometric encoder is a message passing neural network and is trained in advance via a self-supervised learning scheme, in which the geometric encoder learns to reconstruct the original structure of a perturbed complex. This reconstruction objective forces the geometric encoder to capture the intrinsic features underlying the binding interactions between atoms and thus can inform the prediction of binding affinity. The GBT learns, in a supervised way, the mapping from the geometric representations of mutations generated by the trained geometric encoder to the corresponding mutation effects. Compared with traditional methods, we feature the following advantages of GeoPPI: 1) GeoPPI is capable of automatically learning meaningful features of the protein structure for prediction, obviating the need of sophisticated feature engineering. 2) GeoPPI enjoys better generalizability, which results from the geometric encoder that captures the geometric features shared across different protein complexes. 3) GeoPPI is efficient in prediction stage compared to existing methods that rely on computation-heavy biophysical simulations.

In our experiments, we first investigated what the geometric encoder in GeoPPI learns during the self-supervised learning scheme. We found that, without any annotated labels for learning, the geometric encoder captures important knowledge in the protein structure via self-supervised learning, such as the general bond length between atoms, the interface region, the fundamental characteristics of amino acids. Second, we evaluated GeoPPI’s ability of predicting binding affinity changes upon mutations on six benchmark datasets, four for single-point mutations and two for multi-point mutations. GeoPPI obtains the new state-of-the-art performance on all of these datasets with the fastest inference speed, showing its effectiveness and efficiency. Third, we collected several complexes of newly filtered neutralizing antibodies (Abs) bound with the spike glycoprotein of SARS-CoV-2. GeoPPI is able to accurately predict the binding affinity changes between these complexes, even when it is trained with low-order mutants and applied to higher-order mutants. Based on one of these Abs, named C110, GeoPPI locates several residues in its interface where certain mutations can significantly increase the stabilizing effect of the binding with SARS-CoV-2. These results demonstrate that our GeoPPI can serve as a powerful tool for the prediction of binding affinity changes upon mutations and have the potential to be applied in a wide range of tasks, such as designing antibodies with improved binding activity, identifying function-disrupting mutations, and understanding underlying mechanisms of protein biosynthesis.

## Results

### The GeoPPI framework

**Fig 1:**
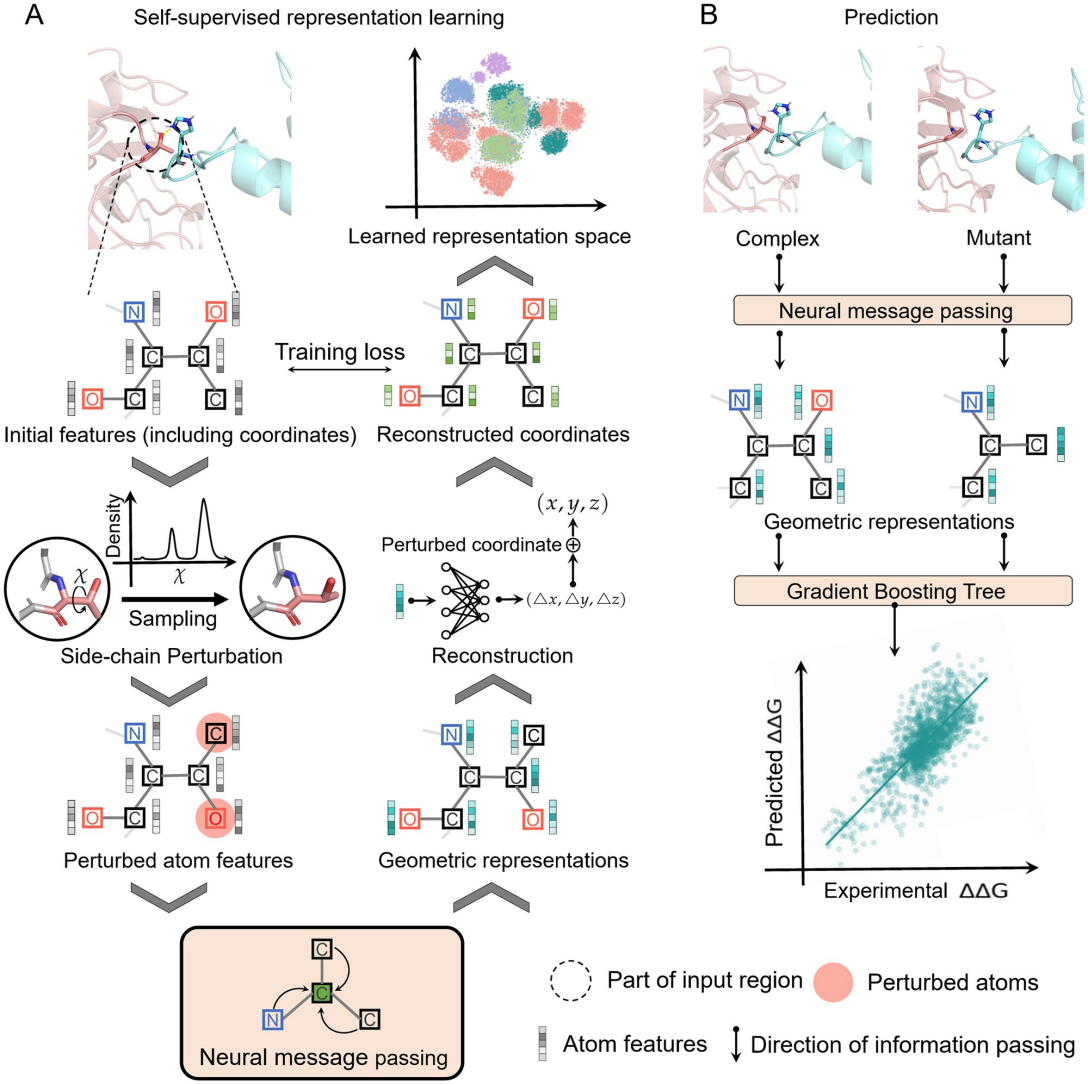
Schematic overview of the GeoPPI pipeline. (A) The self-supervised learning scheme, during which a geometric encoder learns to reconstruct the original structure of a complex given the perturbed one (where the side-chain torsion angles of a residue are randomly sampled). The geometric encoder is a neural network that performs the neural message passing operation on graph structures [19, 20]. The input of the geometric encoder is the graph structure of a complex, where we only consider the atoms that are no more than a predefined distance from either the mutated residues or the interface ones to reduce the computation complexity (Materials and methods). (B) The prediction process of GeoPPI, where the trained geometric encoder produces geometric representations for a given wild-type complex and a mutant, respectively, and a gradient boosting tree (GBT) takes these representations as input to predict the corresponding affinity change.

GeoPPI is a deep learning based framework that uses deep geometric representations of protein complexes to model the effects of mutations on the binding affinity. To achieve both the powerful expressive capacity for geometric structures and the robustness of prediction, GeoPPI sequentially employs two components, namely a geometric encoder (excelling in extracting graphical features) and a gradient-boosting tree (GBT, excelling in avoiding overfitting). The geometric encoder is a graph neural network that performs neural message passing on the neighboring atoms for updating representations of the center atom. It is trained via a novel self-supervised learning scheme to produce deep geometric representations for protein structures. Based on these learned representations of both a complex and its mutant, the GBT learns from the mutation data to predict the corresponding binding affinity change (Fig 1).

Self-supervised learning involves training a model with numerous unlabeled data to obtain deep representations of the input samples [21, 22]. In the self-supervised learning scheme of GeoPPI, the geometric encoder aims to reconstruct the original structure of a complex, given the perturbed one. In particular, we perturb the three-dimensional (3D) coordinates of the side chain of a residue by randomly rotating its side-chain torsion angles. The geometric encoder takes the graph structure of the perturbed complex as input and learns to estimate the coordinate changes during the perturbation and thus reconstructs the original 3D coordinates for the perturbed atoms. This carefully-designed reconstruction task in the self-supervised learning scheme requires the geometric encoder to capture intrinsic patterns underlying the interactions between atoms, providing informative geometric representations for the downstream task (i.e., prediction of binding affinity changes upon mutations). To our best knowledge, GeoPPI is the first method that employs a self-supervised learning scheme to learn representations of the protein structure and utilizes message passing neural networks to model the interactions between atoms in this task, serving as a novel method for estimating the protein-protein binding affinity changes upon mutations.

### GeoPPI captures meaningful patterns in the structure of the protein complex

As our self-supervised learning scheme enforces the geometric encoder to capture general rules in protein structures that occur in nature, we first analyzed what GeoPPI has learned. To this end, we constructed a large-scale training dataset from PDB-BIND [23] and 3DComplex [24] databases for the self-supervised learning. Specifically, we removed the complexes that are identical and similar to the ones in the downstream benchmark mutation datasets and obtained 13590 unlabeled complexes with solved structures as the training dataset (Materials and methods). The complexes in this dataset were randomly split into a training set and a development set. Each complex in the training set was randomly perturbed 2,000 times, used for the training of geometric encoder. The development set is used for validation and analysis. As the binding affinity of two proteins in a complex is largely determined by the interaction strengths between atoms in their interface, below, we tried to test 1) whether the trained geometric encoder in GeoPPI is sensitive to abnormal interactions between atoms; and 2) whether the trained geometric encoder can identify the interface region of a complex; 3) whether the trained geometric encoder can capture the fundamental characteristics (e.g., hydropathy and charge) of individual residues.

**Fig 2:**
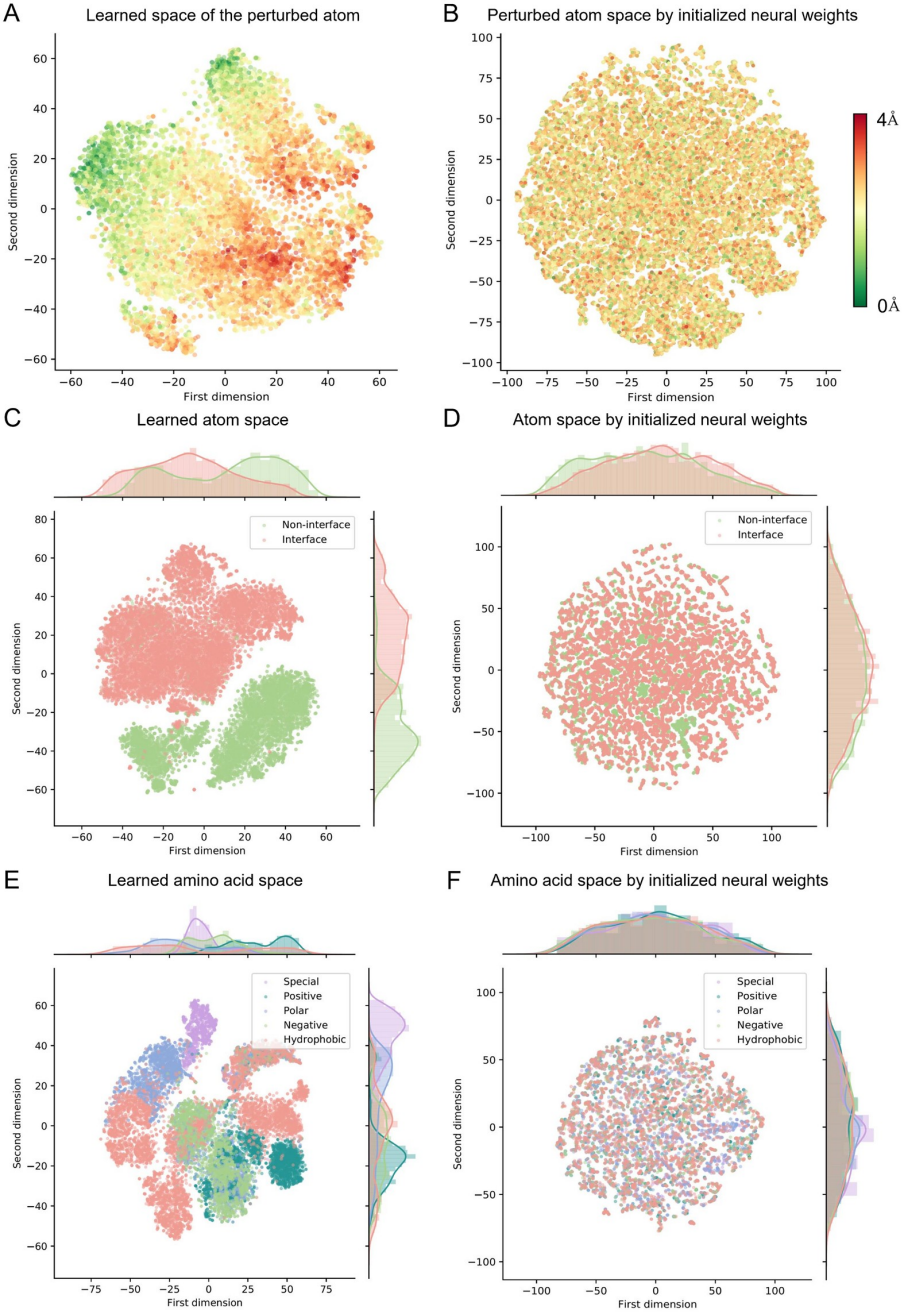
Visualization of the representation space of individual elements in the protein structure. (A) The learned representation space of the atoms with different perturbed distances. (B) The representation space of the perturbed atoms by initialized neural weights (i.e., the weights are not tuned by self-supervised learning). (C) The learned representation space of *α*-carbon atoms, where the color stands for their locations (on or not on the interface) in complexes. (D) The space of *α*-carbon atoms by initialized neural weights. (E) The learned amino acid space, where the color indicates the corresponding group. (F) The amino acid space by initialized neural weights.

To answer the first question, we randomly selected an atom in a complex and perturbed its coordinate within a distance range of 4Å. Then we fed the perturbed structure of the complex into the geometric encoder and obtained the geometric representations of the corresponding perturbed atom. All the complexes in the development set were used and 50 atoms in each complex were randomly selected for this analysis (more atoms did not affect our conclusion). Then, we employed the t-distributed stochastic neighbor embedding (t-SNE) to visualize the distribution of these geometric representations in a low-dimensional space, where the color indicates the perturbed distance (Fig 2A). The t-SNE algorithm is widely used in machine learning to reduce the feature dimension and preserve the most important two components for the input representations [25]. We also performed the same analysis on the geometric encoder that was not trained for comparison (i.e., the neural weights were randomly initialized, Fig 2B). We noticed that, the geometric representations of all the perturbed atoms are scattered uniformly in the space if the geometric encoder is not trained, showing that the geometric encoder initially fails to recognize the abnormal interactions in proteins. After the self-supervised learning scheme, the geometric representations of the perturbed atoms are arranged orderly in terms of the first dimensions. In other words, the most important component of the geometric representations produced by the trained geometric encoder, can clearly indicate the perturbation level of atoms. This comparison demonstrates that the trained geometric encoder can detect the abnormal binding interactions between atoms in a complex. Since the mutations also lead to different conformations in the atom level, the sensitivity to the interactions between atoms is helpful in the prediction of the binding affinity changes upon mutations.

Next, we tried to investigate whether the trained geometric encoder can identify the interface region of the complexes. Concretely, we fed all of the complexes in the development set into the trained geometric encoder separately and obtained the geometric representations of each residue on the interface and those of the non-interface residues. As the *α*-carbon atom is the central point in the backbone of the residue, its geometric features were used to represent the corresponding residue. Then, we employed the t-SNE to visualize the residue space, where the points representing interface residues and non-interface ones are marked with different colors, respectively (Fig 2C and 2D). When we used the geometric encoder that was not trained, the distribution of the residues on the interface is similar to that of non-interface ones. As expected, with the self-supervised learning scheme, the two distributions of the residues on and not on the interface become dramatically different in the two dimensional space reduced by t-SNE. As the input of the geometric encoder includes the information of the interface in the initial atom features (S1 Table), we removed this information from the initial atom features and used the trained geometric encoder to repeat this experiment. Intriguingly, the two distributions of the residues on and not on the interface still present a significant distinction (S1 Fig), indicating that our self-supervised learning scheme enables the geometric encoder to capture the different patterns (e.g., solvent accessibilities [26]) between interface and the non-interface residues.

Finally, in Fig 2E and 2F, we showed the space of the amino acid residues of the complexes in the development set, where the residue is marked with the group of the property it belongs to (i.e., positive, negative, polar, hydrophobic and special-case). The geometric representations of a residue were set to be the summation of geometric representations over all its atoms. Note that both the geometric encoder and the t-SNE algorithm were not informed of the group information. Surprisingly, after the self-supervised learning scheme, the reduced geometric representations of the residues were clustered with respect to the group of property they belong to. This clustering displayed in the main components of the geometric representations demonstrates the trained geometric encoder can learn the physical characteristics of the amino acids from their raw structures, providing the evidence that GeoPPI captures meaningful patterns of the complex structure.

### GeoPPI advances the state of the art in estimating the effects of mutations on binding affinity

We evaluated GeoPPI on six benchmark datasets, namely, the S645, S1131, S4169, S8338, M1101 and M1707 datasets (S2 Table). These datasets have been widely used to test the predictive power of the PPI prediction methods [12, 13, 27–29, 29–35]. The data points in the former four datasets all contain single-point mutations. M1101 contains both single-point and multi-point mutations and M1707 is a multi-point mutation dataset. The digits in the name of each dataset stand for the total number of data points it contains (More information about datasets are provided in Materials and methods).

As some complexes in the above benchmark datasets are highly related (S2 and S3 Figs), machine learning methods tend to be overtrained on these datasets, as evidenced by the drops of the prediction performance when machine learning methods meet unseen structures [31]. Therefore, we adopted a new cross-validation setting where the structures of complexes used in the training and testing are different. Inspired by Shapovalov et al. [36], we used the ECOD (Evolutionary Classification of Domains) homology level [37] to divide the data points to make different folds share no protein domain. The ECOD homology level (“H level”) is a strict similarity criterion since it clusters domains even if they only have distant relationships. Specifically, for a benchmark dataset, we first obtained the classified domains of each complex by uploading the corresponding protein structure to the ECOD server (S3 Table). For the antibody-antigen complexes, we only considered the domains of the antigens since the domains of individual antibodies of a species are usually identical. Then we randomly divided these clusters into five folds according to their domains. Therefore, the data points in different folds share no protein domain. To make the number of data points on individual folds as even as possible, we designed a greedy algorithm for the data division, whose pseudo-code is described in S1 Algorithm. We denote this new cross-validation experiment as the split-by-structure cross-validation (SSCV) to avoid confusion.

We first compared our approach with other competitive methods in SSCV experiments on the single-point mutation datasets (i.e., S645, S1131, S4169 and S8338) (Table 1). We observed FoldX [8] and BeAtMuSic [38] yield the lowest correlations and the highest root-mean-square error (RMSE) on these datasets, indicating the difficulty of gauging the impacts of mutations via empirical energy-based methods. The machine learning methods, such as TopGBT, perform better. This improvement benefits from the fitting process on the part of the dataset, which enables them to learn the mapping from structural features of mutations to the corresponding affinity changes. As for GeoPPI, it outperforms all of the baselines by a large margin on all datasets. In particular, GeoPPI achieves improvements of 45% in terms of Pearson correlation coefficient on S1131 compared with the previous best method TopGBT. TopGBT uses topology-based features to represent the complex, which is not initially designed for representing the interactions between atoms, limiting its predictive power for binding affinity changes upon mutations. By contrast, the self-supervised learning scheme in GeoPPI is built to explicitly learn the interactions between atoms, thus leading to better prediction results. In addition, we tested the prediction performance of GeoPPI on the multi-point mutation datasets (i.e., M1101 and M1707). We noticed the consistent performance gains of GeoPPI over MutaBind2 and FoldX in terms of both Pearson’s correlation and RMSE (Table 2). To situate our work into the current literature, we also reported performances of individual methods in the traditional cross-validation (CV) experiments (where the training and test data points may share similar complexes). We noticed GeoPPI also advances the state of the art in predicting impacts of mutations in the traditional CV (S4 and S5 Tables).

**Table 1:** Comparison of individual methods for the single-point mutations in terms of Pearson correlation coefficient (*R*_*P*_) and root-mean-square error (RMSE) on the S645, S1131, S4169 and S8338 datasets. The methods are evaluated by the split-by-structure cross-validation (SSCV), where ECOD is used in data split to avoid the complexes similar to the training data appearing in the test set. The dash sign indicates the results of the corresponding methods are not available. ^†^: Results were obtained based on the released source code. ^‡^: Results were obtained via the released tool.

Method	S645		S1131		S4169		S8338	
	R_*P*_	RMSE	R_*P*_	RMSE	R_*P*_	RMSE	R_*P*_	RMSE
GeoPPI	**0.51**	**1.70**	**0.58**	**2.01**	**0.52**	**1.48**	**0.68**	**1.49**
TopGBT^†^ [31]	0.39	1.82	0.32	2.31	0.41	1.60	0.61	1.61
TopNetTree^†^ [31]	0.38	1.85	0.29	2.40	0.39	1.65	0.59	1.65
BeAtMuSic^‡^ [38]	0.28	2.03	0.27	2.46	-	-	-	-
FoldX^‡^ [8]	0.30	1.96	0.46	2.18	0.27	2.73	0.44	2.73
Dcomplex [29]	-	-	0.06	-	-	-	-	-

**Table 2:** Comparison of individual methods for the multi-point mutations in SSCV in terms of Pearson correlation coefficient (*R*_*P*_) and root-mean-square error (RMSE) on the M1101 and M1707 datasets. The dash sign indicates the results of the corresponding methods are not available. ^†^: Results were obtained based on the released data. ^‡^: Results were obtained via the released tool.

Method	M1101		M1707	
	R_*P*_	RMSE	R_*P*_	RMSE
GeoPPI	**0.53**	**1.81**	**0.74**	**2.21**
MutaBind2^†^ [15]	-	-	0.72	2.25
Discovery Studio [13]	0.45	-	-	-
FoldX^‡^ [8]	0.34	2.39	0.49	3.02

**Fig 3:**
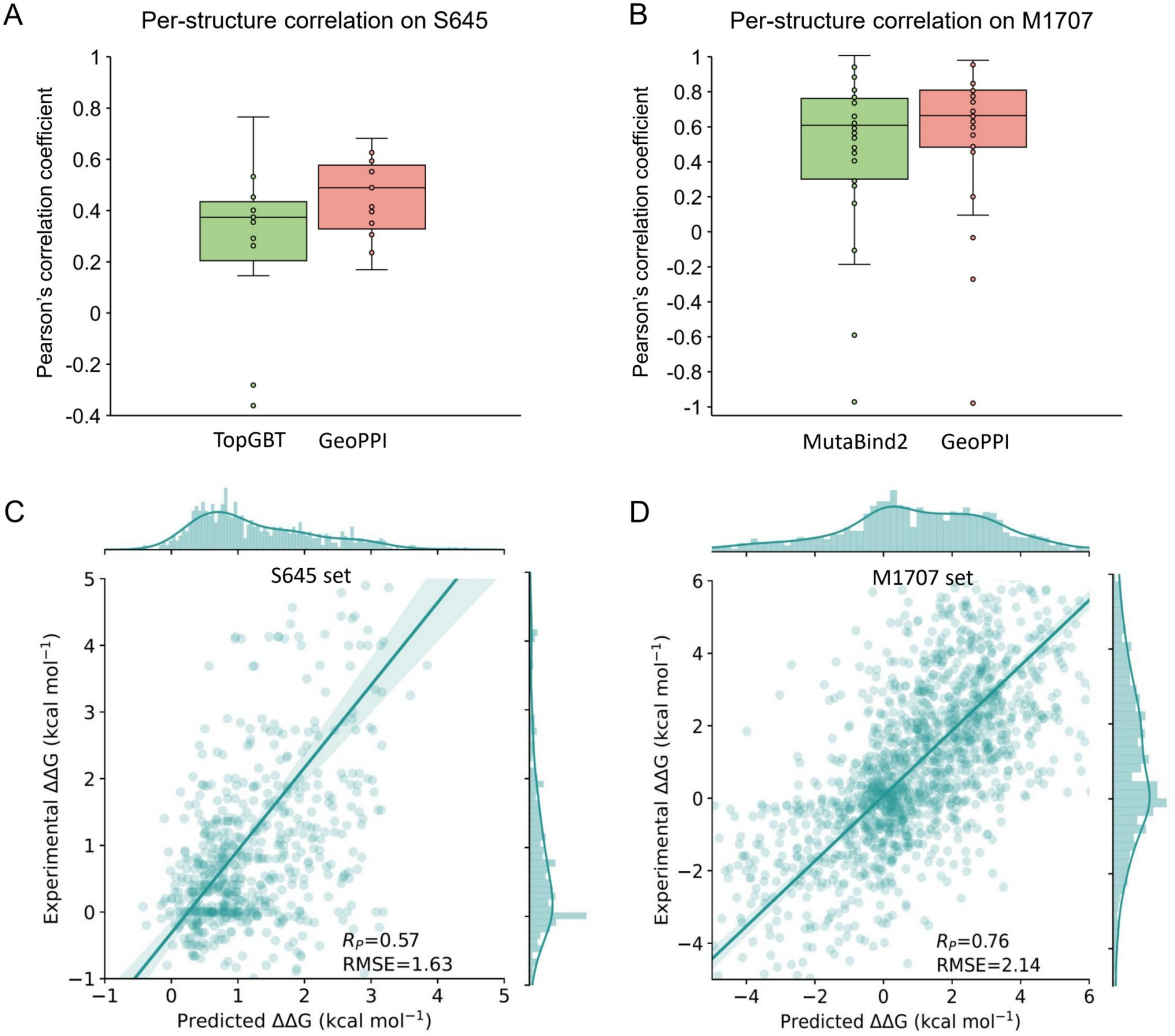
Performance of the prediction models in the leave-one-structure-out cross-validation (CV). (A) Distributions of the per-structure Pearson correlation coefficients of GeoPPI and TopGBT on the S645 dataset. (B) Distributions of the per-structure Pearson correlation coefficients of GeoPPI and MutaBind2 on the M1707 dataset. (C) The experimental values of the affinity changes and those predicted by GeoPPI on S645. (D) The experimental values of the affinity changes and those predicted by GeoPPI on M1707.

In addition to the cross-validation tests as we used previously, here we evaluated the methods using leave-one-structure-out cross-validation (CV) tests on S645 (single-point mutation dataset) and M1707 (multi-point mutation dataset). The leave-one-structure-out CV test involves leaving all the variants of one protein domain as the test set and using the other variants as the training set. By doing this splitting, similar structures in the test set are guaranteed to absent in the training set, which allows us to use the data points of other complexes to estimate the impacts of mutations on previously unseen proteins. In this experiment, we mainly compared GeoPPI with the previous state-of-the-art methods on each benchmark dataset, i.e., TopGBT (on S645, Fig 3A) and MutaBind2 (on M1707, Fig 3B). TopGBT obtains a correlation of 0.39 on S645 while GeoPPI achieves 0.57 (Fig 3C). MutaBind2 obtains a correlation of 0.72 on M1707 while GeoPPI yields 0.76 (Fig 3D). We noticed the increase of prediction performance in leave-one-structure-out CV from SSCV, which is mainly because of the more training data of this experiment. Also, the comparison in per-structure correlations further demonstrates the superiority of our method. Considering that the seven features used in MutaBind2 are manually designed, these features may not comprehensively characterize the impacts of mutations. However, as the features produced by the geometric encoder are learned to describe the differences between the unstable structure and the stable one, leading to the better predictive power of GeoPPI than MutaBind2.

**Fig 4:**
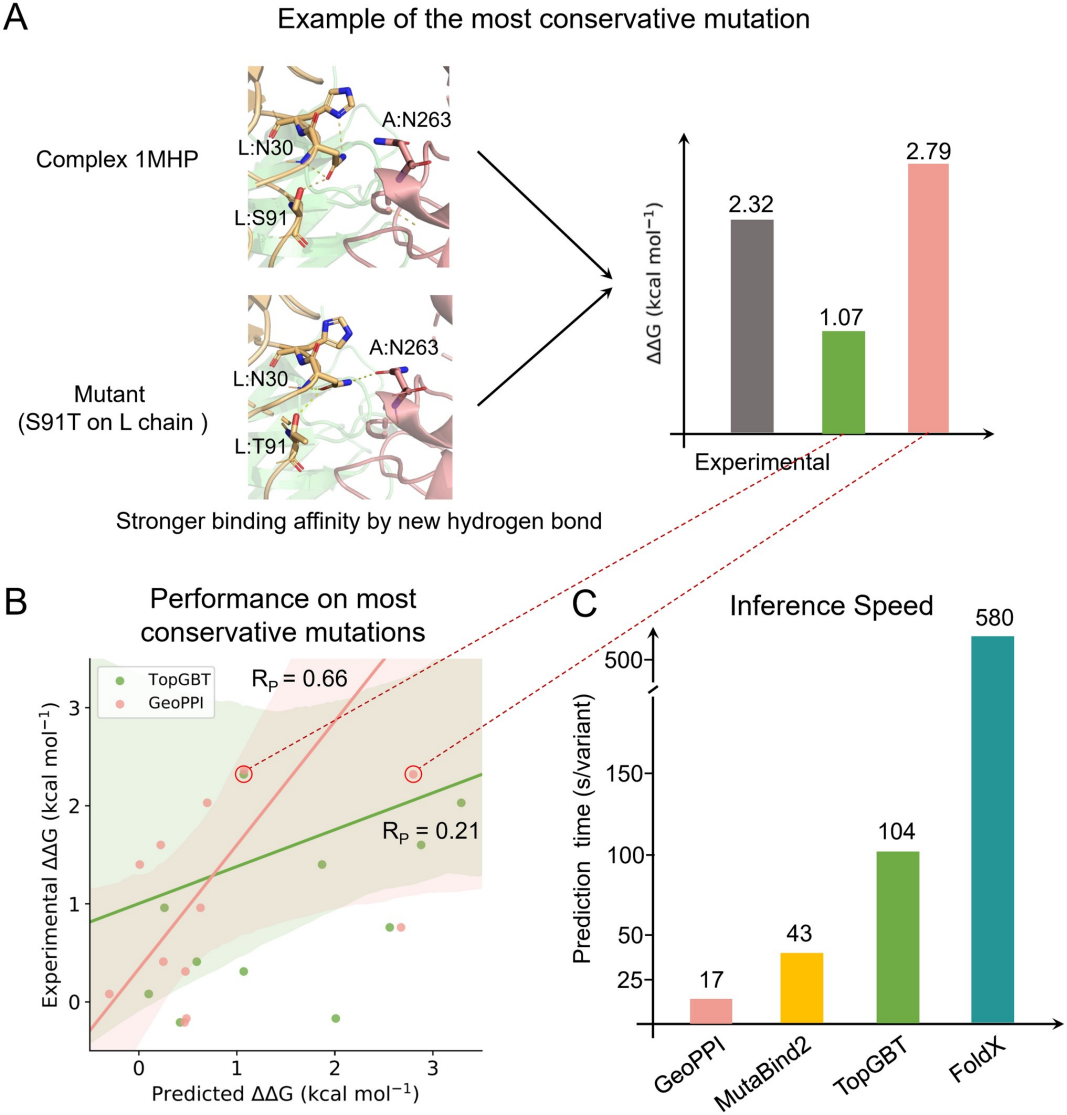
Comparison of GeoPPI with the baseline methods in terms of prediction performance and computational speed. (A) An example of the most conservative mutation and the predicted binding affinity changes by GeoPPI and TopGBT. (B) Prediction performance of GeoPPI and TopGBT on a subset consisting of the most conservative mutations in the S645 dataset. (C) Computational time (second/sample) needed for the prediction of individual methods.

To further understand why GeoPPI can achieve better prediction performance, we analyzed its behavior on the most difficult mutation cases, that is, the most conservative mutations. Here, we defined the most conservative mutation as the substitution that happens between two amino acids that share similar biochemical properties and have only a single atom difference [39]. In spite of the minimum structural changes in these mutations, the binding affinity may also be substantially influenced (Fig 4A). Since non-conservative mutations usually yield larger impacts on function and thus are more likely to be selected against in natural selection due to their deleterious effects, a method that can accurately predict conservative mutations will be more preferred in realistic applications. We collected the predictions of GeoPPI and TopGBT on the most conservative-mutation data points from the S645 dataset and presented them in Fig 4A and 4B and S6 Table. We found that GeoPPI obtains a correlation of 0.66 while TopGBT only yields a correlation of 0.21 on these data points. The unsatisfying results of TopGBT hint that it failed to pay enough attention to this kind of subtle changes in conformations due to its topological abstraction. Conversely, GeoPPI has been shown in Fig 2 to be sensitive on the atom level, which is one of the reasons why it performs much better than TopGBT in this setting. More ablation studies of GeoPPI can be found in S1 Text, S4 Fig and S7 Table.

### GeoPPI shows better prediction generalizability and faster prediction speed

The generalizability of a machine learning method is one of our major concerns since it determines how broadly a machine learning model can be applied in the prediction of the binding affinity of proteins. To this end, we built an independent test dataset to evaluate the generalizability of the models further. Specifically, we took S1131 as the training dataset, as the complexes in S645 are not diverse enough for training and S4169 contains most of the known protein domains among the benchmark datasets (difficult to construct independent test data). We then collected the data points from S1748 (a test dataset used in Zhang et al. [15]) and removed the samples whose complexes are similar to the ones in the S1131 dataset (defined by the ECOD homology level). The filtered dataset contains 641 data points, and thus, is denoted as S641. As the test and training data (i.e., S1131) are from different datasets and share little similarity in the homology level, the test performance can reflect the prediction generalizability of the methods.

The test performances of individual methods are shown in Table 3. We observed that all the methods do not perform well on this test set. Note that the drops from cross-validation performance are not the results of the bias of machine learning models, because FoldX, an empirical energy-based method, also presents a similar decrease. FoldX yields a correlation of 0.46 on S1131 but only obtains 0.16 in this test set, which reflects the challenge of the prediction in this test dataset. However, our model GeoPPI still achieves the highest correlation, showing the generalizability of our method.

**Table 3:** Comparison of prediction performance of GeoPPI with that of different baseline methods on the S641 dataset. In this test, the S1131 dataset is the training dataset of GeoPPI, TopGBT and TopNetTree. Besides the regression performance, a binary classification experiment is conducted to evaluate the ability of classifying the stabilizing and destabilizing mutations in terms of the classification accuracy (ACC), the area under the receiver operating characteristic curve (AUC) and Matthews correlation coefficient (MCC).

Method	Regression		Binary classification		
	R_*P*_	RMSE	ACC	AUC	MCC
GeoPPI	**0.37**	**1.22**	**0.83**	**0.68**	**0.19**
TopGBT [31]	0.32	1.35	0.75	0.66	0.14
TopNetTree [31]	0.27	1.40	0.77	0.63	0.15
BeAtMuSic [38]	0.21	1.41	0.79	0.64	0.13
FoldX [8]	0.16	1.73	0.74	0.64	0.16

Besides the regression performance, we also conducted a binary classification experiment on this test dataset (i.e., S641) to evaluate the ability to classify the stabilizing and destabilizing mutations (Table 3). In particular, we compared individual methods in terms of the classification accuracy, the area under the receiver operating characteristic curve (AUC) and Matthews correlation coefficient (MCC). The AUC score measures the classification ability under different thresholds. The MCC measures robustness on unbalanced datasets. All these metrics show that GeoPPI surpasses other methods in distinguishing the stabilizing and destabilizing mutations, further confirming the superiority of our method in estimating the impacts of mutations.

Due to the considerable number of mutants of a given complex that need to be tested, the speed of predicting the binding affinity changes upon mutations is vital in several applications, such as mutation screening. Here we compared the inference time of each prediction model (Fig 4C and S8 Table). We found that our GeoPPI generally spent 17.2 seconds in predicting the binding affinity change for a single mutant, accelerating the prediction speed of the previously fastest method, i.e., MutaBind2, by 151%.

### GeoPPI accurately predicts effects of mutations of antibodies on their binding affinity with SARS-CoV-2

In this section, we took severe acute respiratory syndrome coronavirus 2 (SARS-CoV-2) as an example to test the realistic utility of our framework. SARS-CoV-2 caused an outbreak of pneumonia as a new world wide pandemic, leading to more than 44 million infection cases and 1 million deaths as of October 29, 2020. SARS-CoV-2 recognizes and attaches to the angiotensin-converting enzyme 2 (ACE2) via the spike (S) glycoprotein when it infects human cells. Antibodies that can effectively block SARS-CoV-2 entry into host cells provide a promising therapy for curing the related diseases. As our framework GeoPPI has shown powerful predictive capacities in various benchmark datasets, here we tested whether GeoPPI can capture the effects of mutations of the antibodies (Abs) on the binding affinities with SARS-CoV-2 and then used GeoPPI to design Abs against SARS-CoV-2.

**Fig 5:**
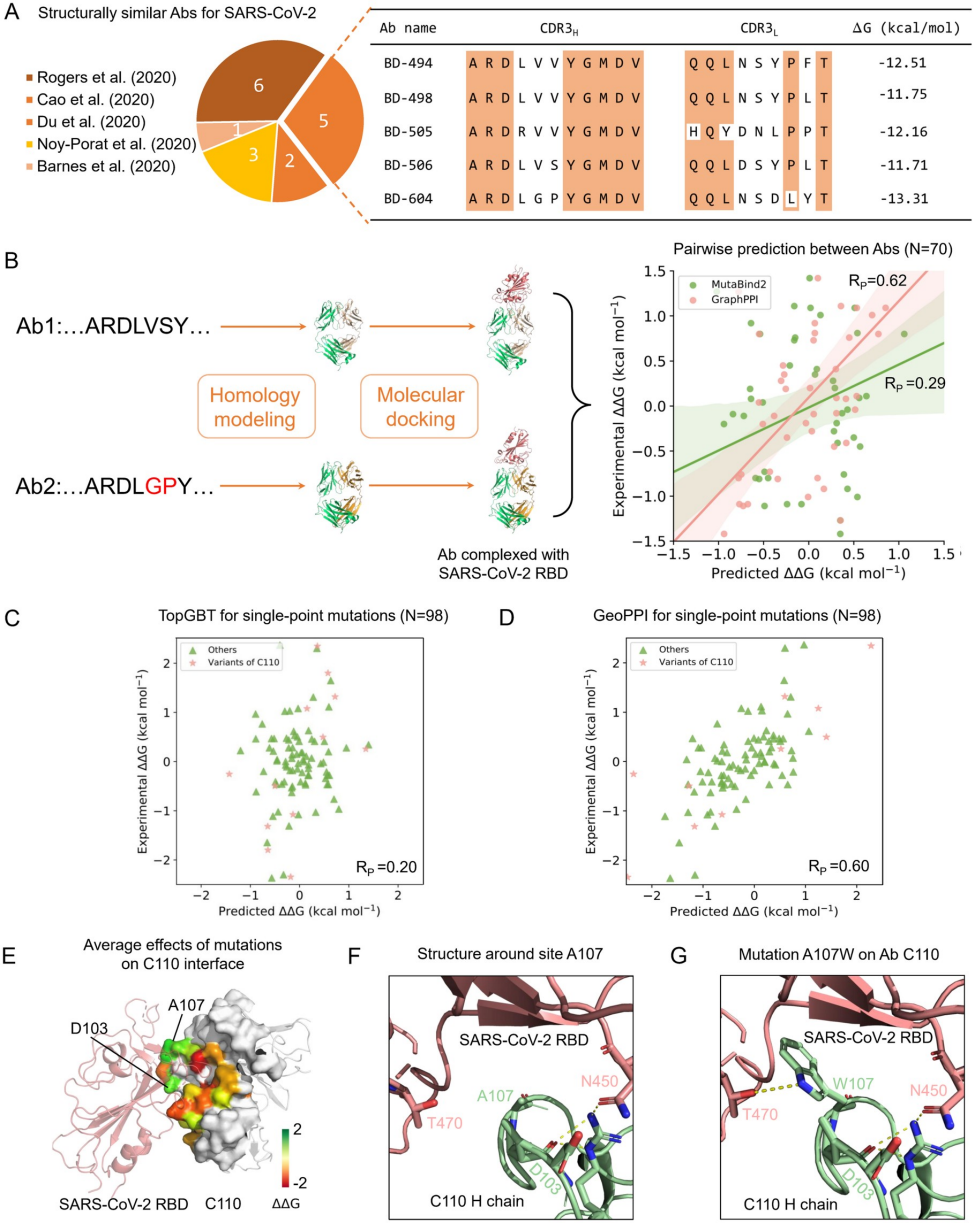
A case study on the antibodies (Abs) that neutralize SARS-CoV-2 by binding with the receptor-binding domain (RBD) of the spike protein. (A) Structurally similar SARS-CoV-2 neutralizing Abs and their CDR3 sequences (S9 Table). (B) Pairwise prediction performance between structurally similar Abs. The structures of these Abs are not solved and approximated by homology modeling. (C and D) Prediction performance of GeoPPI and TopGBT on the single-point mutations of SARS-CoV-2 complexed with individual Abs. This newly collected single-point mutation dataset (S10 Table) contains 98 mutations and corresponding binding affinity changes, including the complexes of SARS-CoV-2 bound to CR3022 [40], C002, C104, C105, C110, C121, C119, C135, C144 [41]. Among them, GeoPPI obtains the highest correlation on the variants of C110. (E) The average predicted affinity changes of the mutations on each residue on the interface of C110 complexed with SARS-CoV-2. (F) The structure around site A107 on C110. (G) The structure around site W107 on C110 with the mutation A107W.

We first constructed a test dataset that contains potent antibodies (Abs) complexed with the SARS-CoV-2 S protein, most of which were recently identified from the convalescent patients [41–43]. These Abs neutralize SARS-CoV-2 by binding with the receptor-binding domain (RBD) of the S protein with different binding strengths. We then filtered 17 structurally similar Abs and used GeoPPI to predict their pairwise affinity changes when binding with SARS-CoV-2 (N = 70, Fig 5A, S9 Table). Most of the structures of these Abs are not determined, making the prediction task more difficult than that on the solved structures. We adopted the Rosetta3 software [44] to perform homology modeling based on the sequences and used ZDOCK software [45] to predict the binding orientations for these Abs, respectively (Materials and methods). Finally, we evaluated the performance of GeoPPI by measuring the difference in the predicted and experimental affinity changes between each pair of these structurally similar Abs when binding to SARS-CoV-2 (Fig 5B).

Considering that the average number of mutations in the training data (i.e., M1707) is 3.3 while the average number of mutations in this Ab dataset is over 10 (S9 Table), the prediction task is quite challenging. However, we noticed that GeoPPI still achieves a strong correlation of 0.62 with the experimental affinity changes. By comparison, MutaBind2 only obtains a weak correlation (i.e., 0.29). These poor results of MutaBind2 were consistent with the previous report that MutaBind2 does not generalize to the cases with large-number mutations [15]. On the contrary, the max-pooling operations may enable GeoPPI to extract the features of the influential mutations and ignore those of marginal mutations, yielding better generalizability to cases with a larger number of mutations. Besides the multi-point mutations, we also collected a single-mutation dataset that contains complexes of several antibodies bound to individual SARS-CoV-2 variants (N = 98, S10 Table). The antibodies involve CR3022 [40], C002, C110, C135, C144 [41], etc. Most of them possess potent neutralizing activities [41, 46]. GeoPPI also achieves significantly better performance than the competitive baseline TopGBT on these test data (Fig 5C and 5D).

As GeoPPI is shown to be capable of accurately predicting the effects of mutations on the Abs complexed with SARS-CoV-2, we tried to leverage GeoPPI to design Abs which can bind with SARS-CoV-2 with better stability (measured by the positive ΔΔ*G*). To this end, we performed a one-step design on the basis of C110, a potent Ab that can recognize both “up” and “down” SARS-CoV-2 RBD conformations [41]. Also, GeoPPI obtains the best performance on the variants of C110 among all of the tested Abs (*R*_*P*_ = 0.83, Fig 5C). Concretely, we performed a full computational mutation scanning on the interface of C110 in complex with the SARS-CoV-2 RBD to investigate which mutations tend to yield higher binding affinities. 19 sites on the interface of C110 were mutated to all the other 19 amino acid types. We thus totally conducted 361 single mutations. Fig 5E illustrates the average effects of mutations on the interface of C110 bound to SARS-CoV-2 RBD. There are two sensitive residues in C110 whose mutations could significantly improve the binding affinity, i.e., A107W and D103Y in the heavy chain. We further studied why the mutation A107W is predicted to have the highest positive impact. We found that it gives rise to a new hydrogen bond between C110 and the SARS-CoV-2 RBD (Fig 5F and 5G), which accounts for the prediction of GeoPPI and thus further confirms the reliability of the prediction by GeoPPI.

Apart from identifying affinity-enhancing mutations for Abs, GeoPPI is also useful to identify mutationally constrained regions on the SARS-CoV-2 surface. As *in vitro* studies suggested that SARS-CoV-2 and SARS-CoV-1 are capable of fixing mutations and thus escaping neutralizing antibodies [47, 48], the antibodies that target mutationally constrained regions on the virus surface can be more effective in curing COVID-19. Therefore, we use the trained GeoPPI (S5 Fig) to perform deep scanning on the surface of the SARS-CoV-2 N-terminal domain (NTD). Intriguingly, we found a large region centered on residue A27 that is mutationally constrained by its binding with ACE2 and is also evolutionarily conserved (S2 Text, S6 Fig). Note that this region has not been targeted by any currently known antibody and might be a promising target that is able to limit the emergence of viral escape mutants.

## Discussion

The determination of protein-protein binding affinity values plays a vital role in understanding the underlying biological phenomena in a cell, such as how missense mutations change the protein-protein binding. The development of machine learning based methods has already demonstrated their promising applications in this problem [8, 10, 15, 31, 49]. In this work, empowered by a self-supervised learning scheme, we have proposed a deep learning based framework, for fast and accurately modeling the binding affinity changes upon amino acid mutations.

The self-supervised learning strategy in the deep learning field derives the supervision signals from the data itself to learn generalized representations of the input, which is useful to the downstream tasks and has been shown to be effective in various fields, such as computer vision [50], natural language [21] and small biological molecule modeling [51]. In particular, Pathak et al. [50] learned the mapping from image to continuous representations by training a convolutional neural network [52] to generate the missing content. Grover et al. [22] obtained the low-dimensional space of node features for node classification by maximizing the likelihood of preserving network neighborhoods of nodes. Different from the previous methods, the self-supervised learning scheme in GeoPPI requires the geometric encoder to reconstruct the coordinates of the perturbed side chains to capture interactions between nodes in the 3D space, which is more challenging than the other problems that are in 1D or 2D spaces (e.g., texts, images, small molecules). Admittedly, the self-supervised learning process usually requires substantial computational resources. In the case of GeoPPI, it takes around 12 days on a GPU (NVIDIA GeForce GTX TITIAN X). Such a time requirement will not be an issue in practice as the training process can be often performed offline.

To our best knowledge, GeoPPI serves as the first study to introduce the self-supervised learning technique for representing the protein structure. As our experiments show that GeoPPI presents better generalizability across the protein structure than previous methods, along this direction, more self-supervised learning strategies can be studied for further improvement of the predictive generalizability of the protein-protein binding affinity. In addition, the learned representations of the protein structure may be also able to benefit other related tasks such as side-chain conformation prediction [53] and macromolecular docking [54]. Overall, we expect our GeoPPI to be applicable and useful to various biological tasks in the future, such as designing antibodies [55], identifying function-disrupting mutations [56], and understanding the underlying mechanisms of protein biosynthesis [57].

## Materials and methods

### Definition of the task of predicting protein-protein binding affinity changes upon mutations

Given the 3D structure of a protein-protein complex, the residue(s) to be mutated and the new amino acid type(s), the goal is to estimate the binding free energy changes (i.e., ΔΔ*G*) between the original complex and the mutant.

$$\begin{array}{cc} & \Delta \Delta G=\Delta G_{\text{wild-type}}-\Delta G_{\text{mutant}}, \end{array}$$ (1)

where

$$\begin{array}{cc} & \Delta G=\text{protein}\;\text{unfolding}\;\text{energy} \end{array}$$ (2)

The positive value of ΔΔ*G* stands for the higher binding affinity between two proteins and the negative value represents the lower binding affinity.

### Datasets

To train and analyze the geometric encoder in the self-supervised learning scheme, we constructed a large-scale training dataset from the PDB-BIND [23] and 3DComplex [24] databases. PDB-BIND is a database that contains 2591 complexes. 3DComplex collects a large number of non-redundant complexes via the hierarchical classification. We adopted a subset of 3DComplex with 40% identity in terms of the protein quaternary structure (denoted by QS40), which contains 33864 complexes. To avoid the leakage of test data points during the training of the geometric encoder, we filtered out the complexes that are identical or similar to the ones in our benchmark datasets from the training dataset. Specifically, we used the ECOD classifier [37] (file: “ecod.develop277.domains.txt”, version: “develop277”) to remove the similar complexes and ensure the training complexes share no ECOD homology domain with the benchmark datasets. However, among the collected data, there are 7199 complexes that have not been covered by ECOD. For these complexes, we leveraged the TMalign software (with a cutoff of 0.5) [58] to further remove the similar complexes. Finally, we took 977 and 12613 complexes from PDB-BIND and 3DComplex databases, respectively. Overall, we used 13590 unlabeled complexes as the training structures of the geometric encoder (10% as the development set).

The six benchmark datasets we used in this work were collected from three protein-protein interaction databases, namely the AB-Bind dataset [34], the SKEMPI dataset [59] and the SKEMPI 2.0 dataset [16]. The AB-Bind dataset contains 1101 data points with experimentally measured binding affinities, also denoted as the M1101 dataset. These data points were derived from studies of 32 antibody-antigen complexes, each comprising 7 to 246 variants (including both single- and multi-point mutations). We also followed Wang et al. [31] to built a subset that only considers single-point mutations in the AB-Bind dataset, called the S645 dataset.

The SKEMPI dataset is a database of 3047 binding free energy changes upon mutations assembled from the scientific literature, for protein-protein heterodimeric complexes with experimentally determined structures [59]. Subsequently, Xiong et al. [30] filtered a subset of 1,131 non-redundant interface single-point mutations from the original SKEMPI dataset, denoted S1131.

As an updated version of the SKEMPI dataset, the SKEMPI 2.0 [16] is composed of 7085 single- or multi-point mutations from 345 complexes. Rodrigues et al. [60] filtered single-point mutations and selected 4,169 variants from 319 different complexes, called the S4169 dataset. The S8338 dataset includes S4169 and all the corresponding reverse mutations. In addition, Zhang et al. [15] collected 1337 variants with multi-point mutations and parts of their reversed mutations to build a multi-point mutation dataset, named the M1707 dataset.

Each data point in the benchmark datasets comprises the 3D structure of a wild-type complex, the residues to be mutated, the new amino acid types and the corresponding binding affinity change. Based on the mutation information, we first used the “build model” function in FoldX [8] to build the 3D structure of the mutated complex. Then we fed the structures of both wild type and the mutant, and mutated positions to GeoPPI for the prediction of affinity changes upon mutations.

### Implementation details of individual modules in GeoPPI

#### Constructing the graph structure of a given complex

To build a graph for a given protein complex, we regard atoms as nodes, and their interactions as edges. We only consider four types of atoms, namely C, O, N, S. For each node *k*, its attributes include element type, the amino acid type, the chain index, the location information (i.e., is on the interface or not), and the three-dimensional coordinate (i.e., $\left(x_{k},y_{k},z_{k}\right)\in R^{3}$). All attributes and their encoding techniques are specified in S1 Table. We concatenate their encodings into a vector. The vector is *D* (i.e., 31) dimensional and used as the initial features of the node. Since the number of atoms in a complex is large, here we only consider the atoms that are near the mutated residues or near the interface of the complex within a distance of 12Å to reduce the computational complexity. The residue whose dASA (changes in accessible surface areas) to a single chain is greater than 1.0 is regarded as the interface site [61]. The features of all the nodes are denoted as $A\in R^{N\times D}$, where *N* stands for the number of considered nodes. As for the edges, if the distance of two nodes is shorter than a threshold (i.e., 3Å), we assume there exists an edge between them. The connected edges on the entire complex are denoted as $E\in R^{N\times N}$, in which the entries are either one or zero. Therefore, for a given complex, its initial graph representations are (***A***, ***E***).

#### Generation of geometric representations by the geometric encoder

In GeoPPI, we propose a geometric encoder to capture the structure of a protein complex at the atom level (Fig 1). The geometric encoder is a message passing neural network and shares the basic idea of the graph attention network (GAT) [19]: for each node, geometric encoder uses the representations of the neighboring nodes to update its representation. But different from GAT, the geometric encoder specifically considers the coordinates in the input vectors when performing the neural message passing operation.

Specifically, given the atom (also called nodes generally) features ***A*** and the edges ***E***, GAT learns to capture the interaction information between atoms. GAT performs a self-attention mechanism on the nodes to indicate the importance of node *j*’s features to node *i*, computed by

$$\begin{array}{c} s_{i,j}=\text{LeakyReLU}(u^{T}[WA_{i}∥WA_{j}]), \end{array}$$ (3)

where ***A***_*i*_, the *i*-th vector in ***A***, stands for the features of the node *i*. ∥ represents concatenation, and LeakyReLU stands for LeakyReLU nonlinear function [62]. $W\in R^{D_{g}\times D}$ and $u\in R^{2D}$ are learnable weights. *D*_*g*_ is the hidden size in GAT.

Different from GAT, the geometric encoder additionally integrates the difference in the coordinates of the two atoms into the self-attention mechanism at the first transformation layer. This is because, in the initial graph features of the complex, the absolute values of three-dimensional coordinates vary a lot across different complexes, the difference between coordinates is more useful. Thus the self-attention mechanism in the geometric encoder is computed by

$$\begin{array}{c} s_{i,j}=\text{LeakyReLU}(u^{T}[WA_{i}∥WA_{j}∥W^{\prime }\left(A_{i}-A_{j}\right)]), \end{array}$$ (4)

where $W^{\prime }\in R^{D_{g}\times D}$ is a learnable weight matrix.

To make coefficients easily comparable across the different nodes adjacent to node *i*, we then normalize them across all choices of *j* using the softmax function,

$$\begin{array}{c} e_{i,j}=\text{softmax}\left(s_{i,j}\right)=\frac{e^{s_{i,j}}}{\sum_{j\in N_{i}}e^{s_{i,j}}}, \end{array}$$ (5)

where $N_{i}$ stands for the set of the neighboring nodes of node *i*. Once obtained, the normalized attention coefficients together with the corresponding atom features are used to apply weighted summation operation, resulting into the updated representations of node *i*, given by

$$\begin{array}{c} H_{i}=\delta \left(\sum_{j\in N_{i}}e_{i,j}WA_{j}\right), \end{array}$$ (6)

where *δ*(⋅) represents the nonlinear function, e.g., ReLU function [62]. The computations of Eqs (4)–(6) form a transformation layer, called the self-attention layer.

The geometric encoder also employs multi-head attention to stabilize the learning process of self-attention, that is, *K* attention mechanisms independently execute the transformation of Eq (6), and then their features are concatenated, resulting in the following feature representations.

$$\begin{array}{c} H_{i}=\text{M-Attention}\left(A,A_{i}\right)={∥}_{k}^{K}\delta \left(\sum_{j\in N_{i}}e_{i,j}^{k}W^{k}A_{j}\right), \end{array}$$ (7)

where $e_{i,j}^{k}$ is normalized attention coefficient computed by the *k*-th attention mechanism, and *W*^*k*^ is the corresponding input linear transformation’s weight. Note that, in this setting, the final returned output *h*_*i*_ will consist of *KD*_*g*_ features (rather than *D*_*g*_) for each node.

To extract a deep representation of the complex structure and increase the expression power of the model, we stacked *L* multi-attention layers.

$$\begin{array}{c} H_{i}^{\left(l+1\right)}=\text{M-Attention}\left(H^{\left(l\right)},H_{i}^{\left(l\right)}\right),l=1,2,\ldots ,L, \end{array}$$ (8)

where ***H***^(*l*)^ stands for the features of all the nodes processed by *l*-th layer of the geometric encoder and $H_{i}^{\left(l\right)}$ indicates processed features for node *i*. Based on the node features processed by the last layer, to further enlarge the receptive field of the transformation for each node and encourage larger values in node features, we also employ max-pooling function to gather the information from the neighboring nodes as part of the final geometric representations of nodes.

$$\begin{array}{cc} g_{i,j} & =\max_{k\in N_{i}}H_{k,j}^{\left(L\right)},\;j\in 1,2,\cdots ,KD_{g}, \end{array}$$ (9)

$$\begin{array}{cc} g_{i} & ={∥}_{j=1}^{j=KD_{g}}\;g_{i,j}∥\;H_{i}^{\left(L\right)}, \end{array}$$ (10)

where $H_{k,j}^{\left(L\right)}$ stands for the entry at the *j*-th dimension of the representations of node *k* obtained at the *L*-th layer.

To summary, given the initial node features ***A*** and the connected edges ***E*** of a complex, the geometric encoder outputs geometric representations $g_{i}\in R^{2KD_{g}}$ for the atom *i* in the complex.

$$\begin{array}{c} G=\left\{g_{i}|i=1,2,\cdots ,N\right\}=\text{GeoEnc}\left(A,E\right), \end{array}$$ (11)

where ***G*** is the set of the geometric representations of all the atoms and GeoEnc stands for the geometric encoder.

### Implementation details of the self-supervised learning scheme

Self-supervised learning has been demonstrated to be powerful in various applications, such as computer vision [50] and natural language processing [21]. The self-supervised learning of graph networks also shows significant performance gains in the task of the prediction of small molecular properties [63]. However, due to the complexity of the dynamics in protein structure, no self-supervised learning scheme has been studied in this field. In this paper, based on the characters of protein conformations, we carefully design a novel self-supervised learning scheme which is specific for the prediction of the affinity changes upon mutation. Generally speaking, in the proposed self-supervised learning scheme of GeoPPI, the geometric encoder aims to reconstruct the original structure of a complex given the perturbed one where the side-chain torsion angles of a residue are randomly sampled. Below, we will elaborate the side-chain perturbation procedure and the reconstruction task.

#### Side-chain perturbation

To produce meaningful perturbations in a given complex, we propose to rotate the side-chain torsion angles of a randomly selected residue. This idea stems from the observation that, for a particular complex, only a few conformations can lead to the lowest free energy. Most of the side-chain perturbations will increase the free energy and make the complex less stable. By reconstructing the original conformations, a model is expected to capture the patterns of the biomolecular interactions between atoms and those between residues in the three-dimensional space.

Formally, let *r* be a certain residue and *ϕ*_*r*_, *ψ*_*r*_ be its two backbone dihedral angles. The perturbed side-chain torsion angles of residue *r* are sampled from the distribution of corresponding side-chain conformations. That is,

$$\begin{array}{c} \chi_{r}∼p\left(\cdot |\phi_{r},\psi_{r},r\right)p\left(r\right), \end{array}$$ (12)

where *p*(⋅|*ϕ*_*r*_, *ψ*_*r*_, *r*) stands for the joint distribution of side-chain torsion angles of the residue *r*, which is approximated by a protein-dependent side-chain rotamer library [64]. *p*(*r*) describes the probability of the residue *r* being selected during the side-chain perturbation. As our downstream task is to model the binding affinity, which is usually characterized by the interface residues of the complex, we set *p*(*r*) to be the uniform distribution over the interface residues and the probabilities of other non-interface residues are zero. Note that individual residues may have different numbers of side-chain torsion angles. For notational simplicity, we use ***χ***_*r*_ to be the set of the torsion angles of the side chain. Taking the glutamic acid for example, there are three torsion angles, that is, ***χ***_*r*_ = (*χ*_*r*,1_, *χ*_*r*,2_, *χ*_*r*,3_).

Based on the sampled side-chain conformations and the coordinates of the backbone of the original residue *r*, we can derive the new coordinate of each atom in residue *r*, which is given by

$$\begin{array}{c} \left({\hat{x}}_{k},{\hat{y}}_{k},{\hat{z}}_{k}\right)=\text{Coordinates}\left(k,\chi_{r},r\right),k\in S\left(r\right), \end{array}$$ (13)

where Coordinates(*k*, ***χ***_*r*_, *r*) stands for the function that yields the coordinates of atom *k* based on the side-chain torsion angles of residue *r*. *S*(*r*) stands for the set of atoms of the side chain of residue *r*. Based on these new coordinates, we can update the matrix of initial node features, denoted by $\hat{A}$, in which the features of other atoms in the graph are kept unchanged. The edges ***E*** of the complex during the side-chain perturbation are also unchanged.

#### Reconstruction

The self-supervised learning scheme requires the geometric encoder in GeoPPI to estimate the original coordinates of the given perturbed complex. However, as the ranges of the coordinates differ a lot in individual complexes, directly predicting the absolute values of the coordinates increases the difficulty of reconstruction. Instead, GeoPPI accomplishes the reconstruction by predicting the difference in coordinates of the atoms in the perturbed residue.

More specifically, we first feed the initial atom features $\hat{A}$ of the perturbed complex into the geometric encoder and obtain the corresponding geometric representations $\hat{G}$ for all the atoms.

$$\begin{array}{c} \hat{G}=\left\{{\hat{g}}_{k}|k=1,2,\ldots ,N\right\}=\text{GeoEnc}\left(\hat{A},E\right). \end{array}$$ (14)

Based on the geometric representations ${\hat{g}}_{k}$ of node *k* generated by the geometric encoder, GeoPPI employs a multi-layer perceptron network (MLP) to predict the change of the coordinate, that is,

$$\begin{array}{cc} \left(△x_{k},△y_{k},△z_{k}\right) & =\text{MLP}({\hat{g}}_{k}). \end{array}$$ (15)

Thus, the predicted coordinate of node *k* can be derived by the summation of the predicted change and the perturbed coordinate.

$$\begin{array}{cc} \left({\tilde{x}}_{k},{\tilde{y}}_{k},{\tilde{z}}_{k}\right) & =\left({\hat{x}}_{k},{\hat{y}}_{k},{\hat{z}}_{k}\right)+\left(△x_{k},△y_{k},△z_{k}\right). \end{array}$$ (16)

The reconstruction loss of GeoPPI is the mean square error between the predicted coordinates and the original coordinates of the perturbed atoms, given by

$$\begin{array}{cc} J & =\frac{1}{\left|S(r)\right|}\sum_{k\in S(r)}\left[{\left(x_{k}-{\tilde{x}}_{k}\right)}^{2}+{\left(y_{k}-{\tilde{y}}_{k}\right)}^{2}+{\left(z_{k}-{\tilde{z}}_{k}\right)}^{2}\right], \end{array}$$ (17)

where |*S*(*r*)| is the cardinality of the set *S*(*r*).

#### Prediction of binding affinity changes upon mutations by gradient-boosting tree

For the prediction of the binding affinity change ΔΔG∈R given the original protein complex and its mutant, GeoPPI integrates the geometric representations ***G*** with a gradient-boosting tree (GBT) [65]. In particular, GeoPPI first leverages the trained geometric encoder to generate features that are expected to represent the affinity change from the original complex to its mutant. For both original complex *o* and its mutant *m*, the learned geometric representations of each atom at the mutated sites (denoted as ***G***_*om*_ and ***G***_*mm*_, respectively) and the learned geometric representations of each atom at the interface sites (denoted as ***G***_*oi*_ and ***G***_*mi*_, respectively) are selected, which are given by

$$\begin{array}{cc} G_{om} & =\left\{g_{k}\right\},k\in S_{om}, \end{array}$$ (18)

$$\begin{array}{cc} G_{mm} & =\left\{g_{k}\right\},k\in S_{mm}, \end{array}$$ (19)

$$\begin{array}{cc} G_{oi} & =\left\{g_{k}\right\},k\in S_{oi}, \end{array}$$ (20)

$$\begin{array}{cc} G_{mi} & =\left\{g_{k}\right\},k\in S_{mi}, \end{array}$$ (21)

where *S*_*om*_ stands for the set of atoms that belongs to the residues to be mutated in the original complex. *S*_*mm*_ stands for the set of atoms that belongs to the residues mutated in the mutant complex. *S*_*oi*_ stands for the set of atoms that belongs to the interface residues in the original complex. *S*_*mi*_ stands for the set of atoms that belongs to the interface residues in the mutant complex.

Due to the specific design in extracting geometric representations in the geometric encoder (such as the ReLU function and Eq (9)), the larger values of features represent higher importance. Therefore, for each collection of the geometric representations (namely ***G***_*om*_, ***G***_*mm*_, ***G***_*oi*_ and ***G***_*mi*_), we use max-pooling and mean-pooling operations to obtain their max values and mean values at each dimension over the selected atoms, that is,

$$\begin{array}{cc} & F_{n}=\left[\text{max-pooling}\left(G_{n}\right),\text{mean-pooling}\left(G_{n}\right)\right], \end{array}$$ (22)

$$\begin{array}{cc} & \text{max-pooling}{\left(G_{n}\right)}_{d}=\max\left\{g_{k,d},k\in S_{n}\right\}, \end{array}$$ (23)

$$\begin{array}{cc} & \text{mean-pooling}{\left(G_{n}\right)}_{d}=\text{mean}\left\{g_{k,d},k\in S_{n}\right\}, \end{array}$$ (24)

$$\begin{array}{cc} & n\in \left\{om,mm,oi,mi\right\},d\in 1,2,\cdots ,2KD_{g}, \end{array}$$ (25)

where *d* denotes the dimension index of the learned representations.

We feed these processed geometric representations into a gradient boosting tree (GBT) to rank their importance and use the top *N*_F_ features to accomplish the prediction of the binding affinity changes (i.e., ΔΔ*G*) upon mutations.

$$\begin{array}{c} \Delta \Delta G=\text{GBT}(\left[F_{om},F_{mm},F_{xi},F_{mi}\right],N_{\text{F}}). \end{array}$$ (26)

### Training techniques

For each complex in the training dataset of geometric encoder, we perturbed the structure by randomly selecting a residue and randomly sampling its side-chain torsion angles based on the observed distribution [64]. We repeated the side-chain perturbation 2,000 times for each complex, resulting in 27,180,000 data points in the dataset. During the training, the standard batch gradient descent method [66, 67] with the error back-propagation algorithm was performed using the Adam algorithm with the default settings [68]. The best hyperparameters of GeoPPI were calibrated through a grid search procedure on the development set (S11 Table).

As for the learning of GBT for each dataset, we trained the GBT using the training data of each fold in the cross-validation tests. The selection of the hyper-parameters in all the experiments of this paper was also based on the training data. Taking a fold of the cross-validation experiment as an example, we held out 10% of the training data as the development data; We chose the hyper-parameters of the GBT that yield the highest performance on the development data. The hyperparameters of GBT involve *N*_F_, *N*_estimator_ and *D*_max_, which stand for number of selected features, the number of regression estimators and the maximum depth of the individual estimators, respectively. The hyper-parameters were chosen from *N*_F_ ∈ {100, 120, 140}, *N*_estimator_ ∈ {3, 4, 5} × 10^4^ and *D*_max_ ∈ {4, 6, 8}. Based on the chosen hyper-parameters, we trained the GBT on the training data and tested the GBT on the validation data.

GeoPPI was implemented based on the PyTorch library 1.7.0 [69] and the scikit-learn library 0.24.1 [70]. The NVIDIA GeForce GTX TITAN X GPU was used to speed up the training process.

### Implementation details of the tests on SARS-CoV-2 related datasets

To build the dataset for pairwise affinity prediction between Abs, we collected 17 structurally similar Abs that can neutralize SARS-CoV-2 from recent studies [41–43, 71, 72]. Their binding affinities with SARS-CoV-2 were measured by surface plasmon resonance (SPR). However, their structures were not solved. For each of these Abs, we first selected some Ab templates that share high homology with it and leveraged the “comparative modeling” function in the Rosetta3 software [44] to obtain some candidate structures. The structure with the highest score generated by Rosetta3 was chosen. The selected templates were listed in S9 Table. Then we adopted ZDOCK software [45] to predict the orientation of the SARS-CoV-2 RBD to the Ab of interest. Finally, we selected the mutations between two Abs where the numbers of mutated points are less than 20 to construct the dataset. Before the prediction methods (i.e., GeoPPI and MutaBind2) were evaluated on this dataset, they were trained on the M1707 dataset, a high-quality multi-point mutation dataset.

As for the single-point mutation dataset for SARS-CoV-2, we collected 9 Abs, each complexed with SARS-CoV-2 RBD separately. These Abs are CR3022, C002, C104, C105, C110, C121, C119, C135 and C144. The structures of these Abs are available. The effects of mutations on SARS-CoV-2 on the binding affinity with these Abs were measured by Barnes et al. [41] and Wu et al. [40]. We also included their reversed mutations in this dataset, leading to a total of 98 data points. Before this test, we trained GeoPPI and the baseline TopGBT in advance on the S645 dataset where the training data are antibody-antigen complexes.

### Implementation details of deep mutational scanning on the SARS-CoV-2 NTD

The structure of SARS-CoV-2 S1 subunit complexed with ACE2 (denoted by S1-ACE2) is not directly available. To estimate the effects of mutations of the SARS-CoV-2 NTD on the binding affinity with ACE2, there is a need to build its structure. We aligned the structure of S protein of SARS-CoV-2 (PDB ID: 7c2l) and that of SARS-CoV-2 RBD bound with ACE2 (PDB ID: 6m0j) to obtain an integrated structure of S1-ACE2. During the deep mutational scanning on the SARS-CoV-2 NTD (total 312 sites), each residue was mutated to the other 19 amino acid types, resulting in 5928 single-point mutations. GeoPPI was trained on the data collected from Starr et al. [73] (S2 Text). For the prediction of the effect of each mutation, we first used “buildmodel” function in FoldX to build the 3D structure of the mutant based on the structure of the complex S1-ACE2, and then fed the structures of both wild type and the mutant into GeoPPI to obtain the corresponding predicted value.

## Supporting information

S1 Text Ablation study. (PDF)

S2 Text GeoPPI identifies mutationally constrained regions in the SARS-CoV-2 spike N-terminal domain. (PDF)

S1 Fig Visualization of the geometric representations of the *α*-carbon atoms by t-SNE. The geometric representations of the *α*-carbon atoms on and not on the interface were produced by the trained geometric encoder. In the input of the geometric encoder, the location information of the initial atom features was masked to zeros.
(PDF)

S2 Fig The similarities between arbitrary two complexes in the S645 dataset measured by the TMalgin software. (PDF)

S3 Fig The similarities between arbitrary two complexes in the M1707 dataset measured by the TMalgin software. (PDF)

S4 Fig The prediction performance of GeoPPI with different learning strategies and transformation layers (i.e., the geometric encoder and MLP) on the split-by-structure CV. “GeoEnc” stands for the geometric encoder. To test the effectiveness of the geometric encoder, we built a control framework that uses a multiple layer perceptron (MLP) to replace the geometric encoder. More specifically, each multi-attention transformation layer (Eq (8)) was replaced by an MLP layer. The main difference between the geometric encoder and MLP lies in the way of processing the information of neighboring nodes. For a node in the graph structure, MLP updates the representations based on its own representations, while the geometric encoder can aggregate the information from the neighboring nodes for the update.
(PDF)

S5 Fig The structure of the spike monomer of SARS-CoV-2 complexed with 4A8 and the performance of individual methods in the S3647 dataset. (A) The structure of the spike monomer of SARS-CoV-2 complexed with 4A8 (from PDB ID: 7c2l). (B) The performance of GeoPPI in the S3647 dataset in terms of the ten-fold CV test. (C) The performance of TopGBT in the S3647 dataset in terms of the ten-fold CV test. In the S3647 dataset, the binding affinity (Δ*G*) is measured by the apparent dissociation constant log_10_(*K*_*D*,*app*_). The Pearson correlation coefficient and root mean square error for each method are shown in the upper left corner of the subfigure.
(PDF)

S6 Fig Deep mutational scanning on all the sites of the SARS-CoV-2 NTD. (A) Heatmaps representing how single-point mutations on the SARS-CoV-2 NTD impact the binding affinity with ACE2. The mutation that leads to an increase of binding affinity was circled in the green color. (B) The mutational constraint of the epitope of 4A8, an antibody targeting the SARS-CoV-2 NTD. The surface of NTD is colored according to the average mutational effects on the binding affinity with ACE2. (C) Identification of a patch of mutational constraint surrounding NTD residue A27. (D) The comparison of evolutionary conservation between the epitope of 4A8 and the newly identified A27 patch. The evolutionary conservation profiles of residues were calculated by ConSurf Database [74] based on the sequence alignment among 37 SARS-CoV-2 related sarbecoviruses summarized in Starr et al. [73].
(TIF)

S1 Table Node features and corresponding encoding methods. (PDF)

S2 Table Experimental benchmark datasets. (PDF)

S3 Table The protein domains for individual complexes in the benchmark datasets measured by ECOD. (XLSX)

S4 Table Comparison of Pearson correlation coefficient of various methods for the single-point mutations in ten-fold CV on the S645, S1131, S4169, S4191 and S8338 datasets. *: Results are obtained based on the released source code.
(PDF)

S5 Table Comparison of Pearson correlation coefficient of various methods for the multi-point mutations on the M1101 and M1707 datasets. The performance of GeoPPI and MutaBind2 on the M1101 dataset was obtained by the ten-fold CV. To have a fair comparison with the MutaBind2 on M1707, GeoPPI and FoldX were evaluated with the two-fold cross validation test.
(PDF)

S6 Table The experimental and predicted binding affinity changes (kcal/mol) upon the most conservative mutations on the S645 dataset, including the mutations from D to E, S to T, V to I, F to Y and their reversed mutations. (PDF)

S7 Table Analysis of the fold number in the split-by-structure cross validation. The analysis was conducted on the S4169 dataset.
(PDF)

S8 Table Computational time (s/sample) needed for the prediction of each method. The measurement was conducted using 1000 single-point mutations from a complex with 350 residues. Molecular dynamics with FoldX (MD-FoldX) and coarse-grained-umbrella sampling simulations (CG-US) are two molecular modeling methods for estimating the affinity changes upon mutations [75]. The computational time of TopGBT is obtained by running its source code. The test was conducted in the single CPU (Intel Core i7–4790K) or single GPU (NVIDIA GeForce GTX TITIAN X GPU) setting. ^†^: Results were quoted from Patel et al. [75]. ^‡^: Results were quoted from Zhang et al. [15].
(PDF)

S9 Table Sequences of the structurally similar SARS-CoV-2 neutralizing Abs. (XLSX)

S10 Table The data points of the SARS-CoV-2 single-point mutation dataset. (XLSX)

S11 Table The selected hyperparameters of GeoPPI. The hyperparameters in the geometric encoder include the hidden size *D*_*g*_, the number of attention heads *K*, number of hidden layers *L*. We applied a coarse grid search approach over *D*_*g*_ ∈ {128, 256, 512}, *K* ∈ {2, 4, 6, 8, 16}, *L* ∈ {1, 2, 3, 4, 5} on the development set of the self-supervised learning dataset to select the best settings of these hyperparameters.
(PDF)

S1 Algorithm Greedy algorithm for data division (python style). (PDF)
