# Supplementary material for: Deep geometric representations for modeling effects of mutations on protein-protein binding affinity
Source: PLoS Comput Biol. 2021 Aug 4;17(8):e1009284. doi: 10.1371/journal.pcbi.1009284 (PMC8366979; doi:10.1371/journal.pcbi.1009284)
Supplement: S1 Text — (PDF) [file pcbi.1009284.s001.pdf]

In our experiments, we have also conducted a series of ablation studies to investigate the influences of different design choices of GeoPPI on the prediction performance and the influences of the SSCV fold number on the prediction performance.

We first compared the difference in the performance of GeoPPI with and without the self-supervised learning scheme on split-by-structure CV tests. As shown in S4 Fig, without a self-supervised learning scheme, GeoPPI shows a significant decrease in prediction performance. This result shows that the well-designed self-supervised learning scheme greatly improves the predictive power of GeoPPI. Next, the ablation study on a key component in GeoPPI, i.e., the geometric encoder, further confirms that it plays a critical role in representing the complex structures and is superior to other neural networks (e.g., the multiple layer perceptron, S4 Fig).

It is widely accepted that the results should not vary much between the CV experiments with different fold numbers if bias was not introduced. To validate this, we also conducted another ablation study to investigate the influence of the fold number in cross-validation on a relatively large dataset, i.e., S4169. In particular, we evaluated GeoPPI with nine split-by-structure cross-validations (SSCV), with different fold numbers ranging from 2 to 10. The performances on individual SSCV are shown in S7 Table. We observed that GeoPPI obtains similar performances on different SSCV fold numbers, which shows that our SSCV experiments are not biased and offer a robust way to evaluate the prediction capacity of the existing methods. We also noticed that GeoPPI performs slightly better on the SSCV with a larger fold number. It is reasonable because a cross-validation experiment with a larger fold size has more training data and fewer test data, resulting in better results [1].

References

[1] Kohavi R, et al. A study of cross-validation and bootstrap for accuracy estimation and model selection. In: IJCAI. vol. 14. Montreal, Canada; 1995. p. 1137–1145.
