# Supplementary material for: Deep geometric representations for modeling effects of mutations on protein-protein binding affinity
Source: PLoS Comput Biol. 2021 Aug 4;17(8):e1009284. doi: 10.1371/journal.pcbi.1009284 (PMC8366979; doi:10.1371/journal.pcbi.1009284)
Supplement: S2 Text — (PDF) [file pcbi.1009284.s002.pdf]

Apart from the robust prediction performance, GeoPPI is also useful in the following two aspects. On the one hand, finding ACE2-affinity-enhancing mutations in SARS-CoV-2 is critical for viral surveillance; On the other hand, identifying the mutationally constrained regions on the surface of the SARS-CoV-2 could reveal the desirable targets for vaccines and antibody-based therapeutics. The S protein in SARS-CoV-2 plays a critical role in recognizing angiotensin-converting enzyme 2 (ACE2) when it infects human cells. The S1 subunit in the S protein is responsible for receptor recognition, which can be further divided into an N-terminal domain (NTD) and a receptor-binding domain (RBD, S5A Fig). Liu et al. [1] showed both of these regions were highly immunogenic. The effects of mutations on RBD have been widely studied [2, 3] but NTD still lacks sufficient attention. Here we mainly focus on the SARS-CoV-2 NTD in terms of the aforementioned two aspects.

To identify the ACE2-affinity-enhancing mutations, we first built a training dataset to obtain the best prediction capacity. The training dataset contains 3647 mutations of SARS-CoV-2 (denoted by S3647). The effects of these mutations on the binding affinity with ACE2 were measured by Starr et al. [2]. Each affinity change in the dataset was the average over the values determined from two independent library replicates, measured by the change in the logarithm of the apparent dissociation constant $\Delta \log_{10}(K_{D,app})$. We first tested the prediction performance of GeoPPI and that of TopGBT on this dataset in ten-fold CV. As shown in S5B and S5C Fig, GeoPPI consistently outperforms TopGBT in terms of both Pearson correlation and RMSE. Meanwhile, the correlation between the two replicates was 0.95, indicating the upper bound of the performance that a machine learning method can achieve. Considering that GeoPPI obtains a correlation of 0.88, our test results demonstrated that GeoPPI is able to capture the effects of mutations on SARS-CoV-2 to a large extent. Then, we used all the data points in the S3647 dataset to train GeoPPI and employed GeoPPI to perform deep mutational scanning to estimate how all the mutations on the SARS-CoV-2 NTD impact its binding affinity with ACE2 (Materials and methods). Concretely, each site in NTD (total 312 residues) was mutated to the other 19 amino acid types, resulting in 5928 single-point mutations (S6A Fig). We observed that most of the mutations on the SARS-CoV-2 NTD turn out to have negative effects on the binding affinity with ACE2. There are also a handful of sites that are tolerant of mutations (e.g., 205-206, 296-298). Only one mutation, S205A, is predicted to enhance the binding affinity with ACE2 (predicted $\Delta\Delta G = 0.33$). Therefore, it may have a higher probability to be selected in future SARS-CoV-2 pandemic isolates.

We further analyzed the effects of mutations on each site of the NTD surface to search for mutationally constrained regions. Generally speaking, there exist a number of sites that are mutationally constrained (reflected by the red color in S6B Fig); The majority of them are scattered across the NTD surface and some of them form several hotpot regions of different sizes. Based on these results, the epitopes of known antibodies can be analyzed. Chi et al. [4] identified an NTD-targeting antibody, 4A8, that potently neutralizes SARS-CoV-2 (S5A Fig). However, several binding sites (e.g., sites 147-150 and 247) on its epitope can be mutated with little binding affinity loss (i.e., not strongly constrained), revealing the possibility of viral escape. In addition, we identified a large region centered on residue A27 that is mutationally constrained by its binding with ACE2 (S6C Fig). Also, the further comparison with the epitope of 4A8 hints that the newly identified A27 patch is more evolutionarily conserved (S6D Fig). Note that this region has not been targeted by any currently known antibody and might be a promising target that is able to limit the emergence of viral escape mutants.

References

[1] Liu L, Wang P, Nair MS, Yu J, Rapp M, Wang Q, et al. Potent neutralizing antibodies against multiple epitopes on SARS-CoV-2 spike. Nature. 2020;584(7821):450–456.

[2] Starr TN, Greaney AJ, Hilton SK, Crawford KH, Navarro MJ, Bowen JE, et al. Deep mutational scanning of SARS-CoV-2 receptor binding domain reveals constraints on folding and ACE2 binding. Cell. 2020;182(5):1295–1310.

[3] Chen J, Wang R, Wang M, Wei GW. Mutations Strengthened SARS-CoV-2 Infectivity. Journal of Molecular Biology. 2020;doi:https://doi.org/10.1016/j.jmb.2020.07.009.

[4] Chi X, Yan R, Zhang J, Zhang G, Zhang Y, Hao M, et al. A neutralizing human antibody binds to the N-terminal domain of the Spike protein of SARS-CoV-2. Science. 2020;369(6504):650–655. doi:10.1126/science.abc6952.
