# Supplementary figures and images for: Deep geometric representations for modeling effects of mutations on protein-protein binding affinity
Source: PLoS Comput Biol. 2021 Aug 4;17(8):e1009284. doi: 10.1371/journal.pcbi.1009284 (PMC8366979; doi:10.1371/journal.pcbi.1009284)

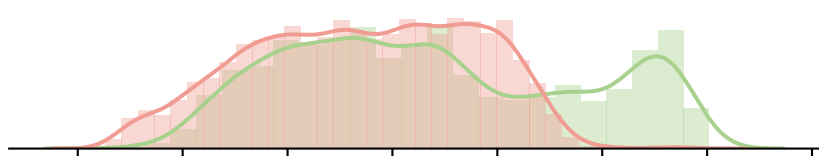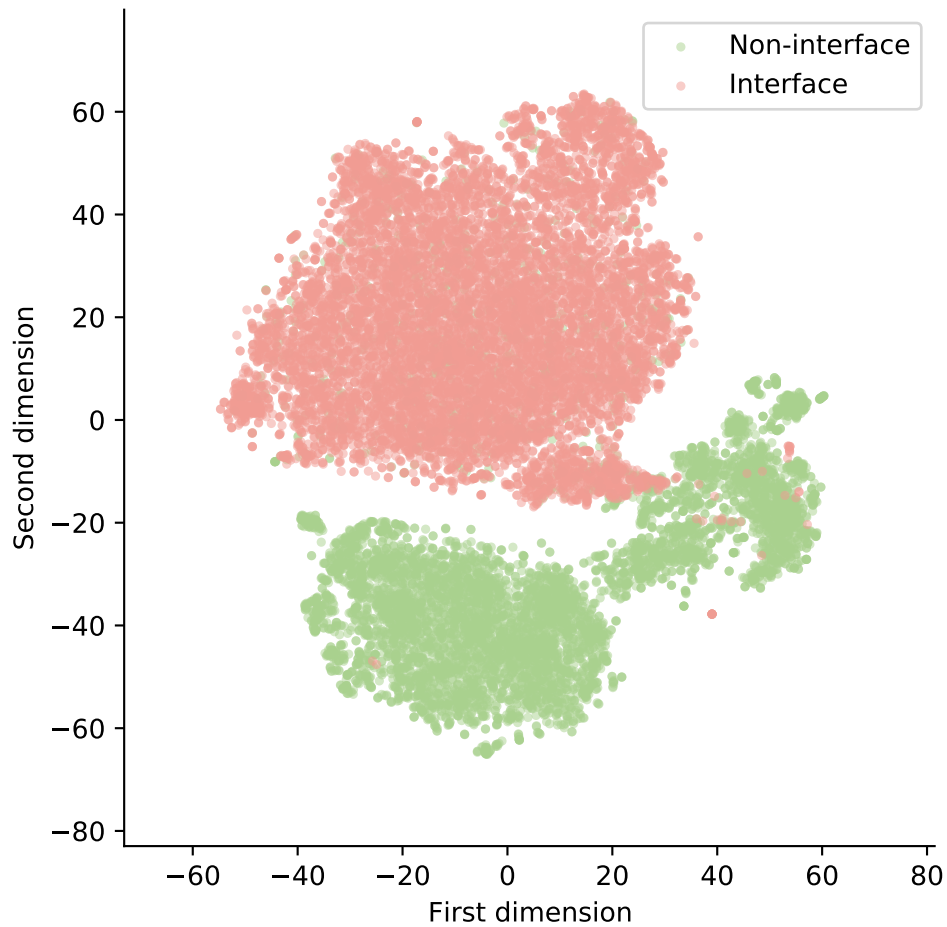

Supplement: S1 Fig — The geometric representations of the α-carbon atoms on and not on the interface were produced by the trained geometric encoder. In the input of the geometric encoder, the location information of the initial atom features was masked to zeros. (PDF) [file pcbi.1009284.s003.pdf]

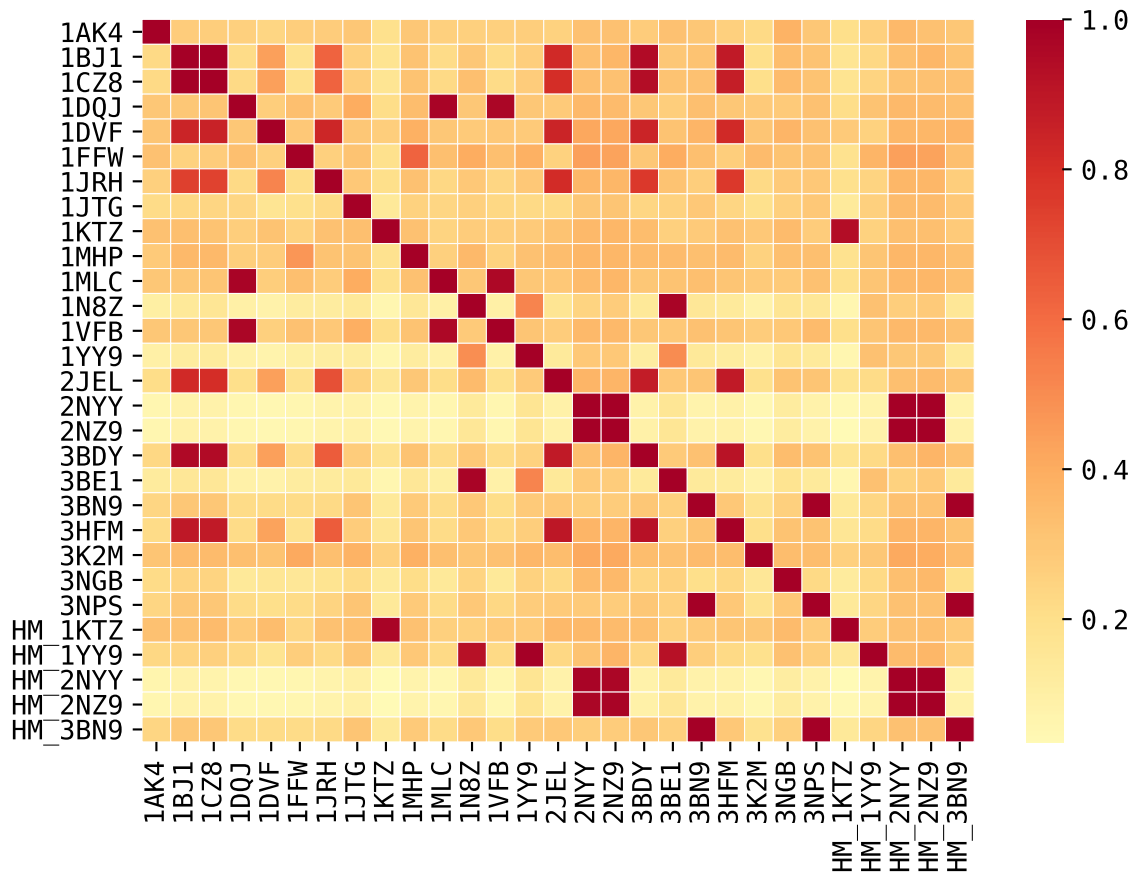

Supplement: S2 Fig — (PDF) [file pcbi.1009284.s004.pdf]

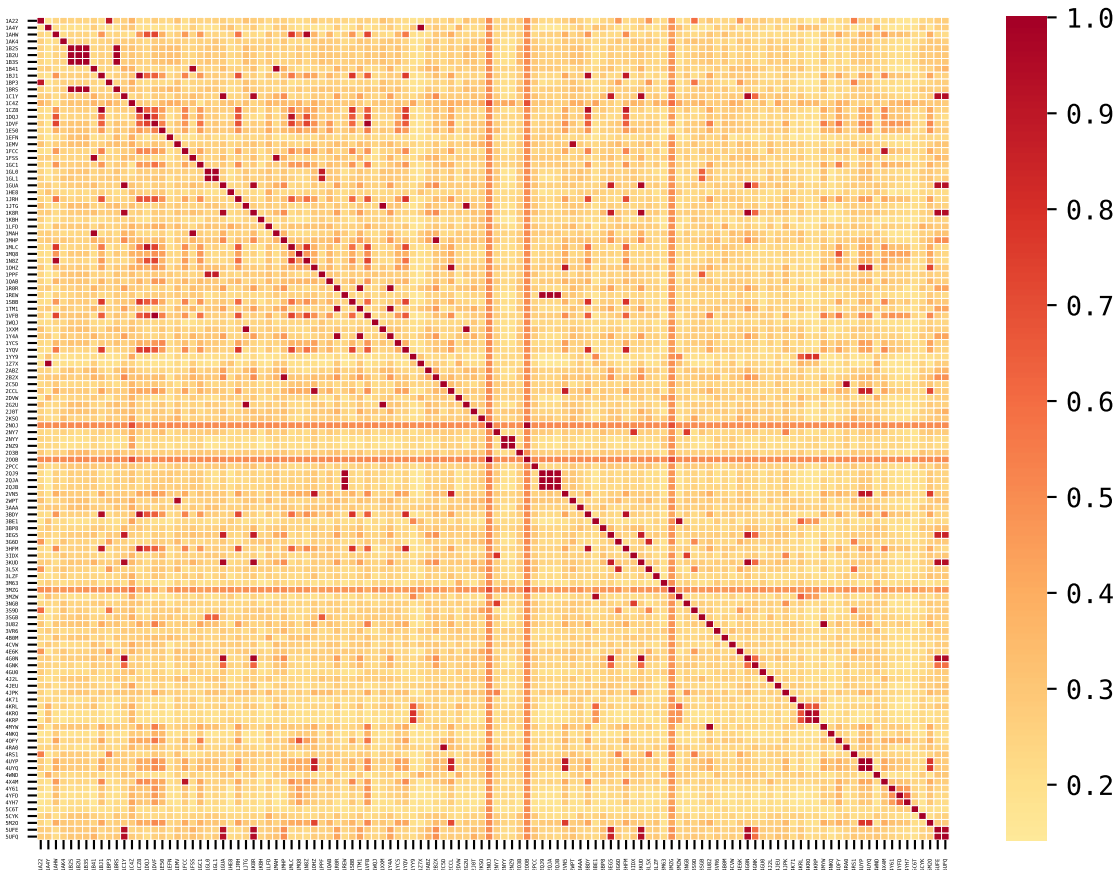

Supplement: S3 Fig — (PDF) [file pcbi.1009284.s005.pdf]

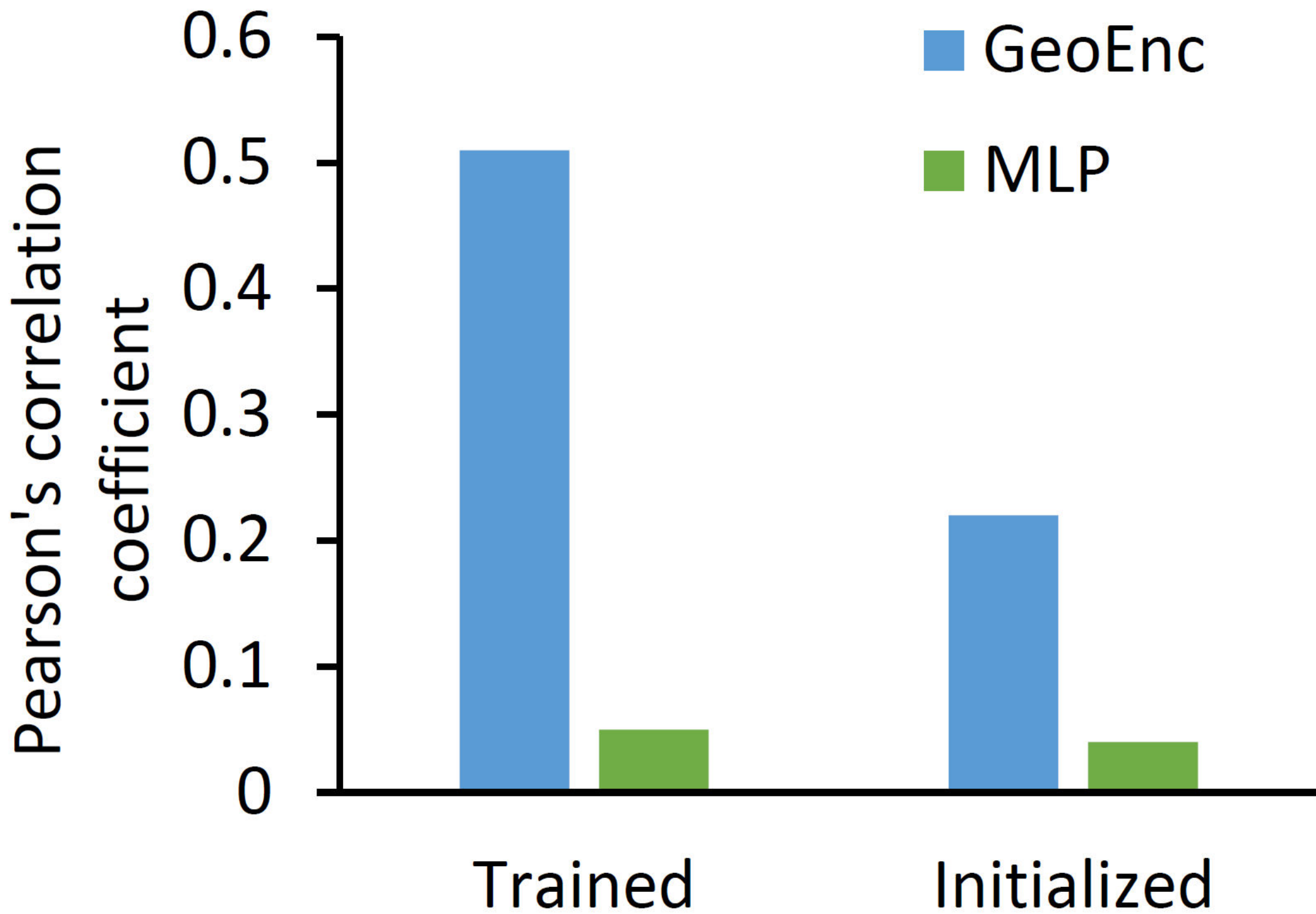

Supplement: S4 Fig — “GeoEnc” stands for the geometric encoder. To test the effectiveness of the geometric encoder, we built a control framework that uses a multiple layer perceptron (MLP) to replace the geometric encoder. More specifically, each multi-attention transformation layer (Eq (8)) was replaced by an MLP layer. The main difference between the geometric encoder and MLP lies in the way of processing the information of neighboring nodes. For a node in the graph structure, MLP updates the representations based on its own representations, while the geometric encoder can aggregate the information from the neighboring nodes for the update. (PDF) [file pcbi.1009284.s006.pdf]

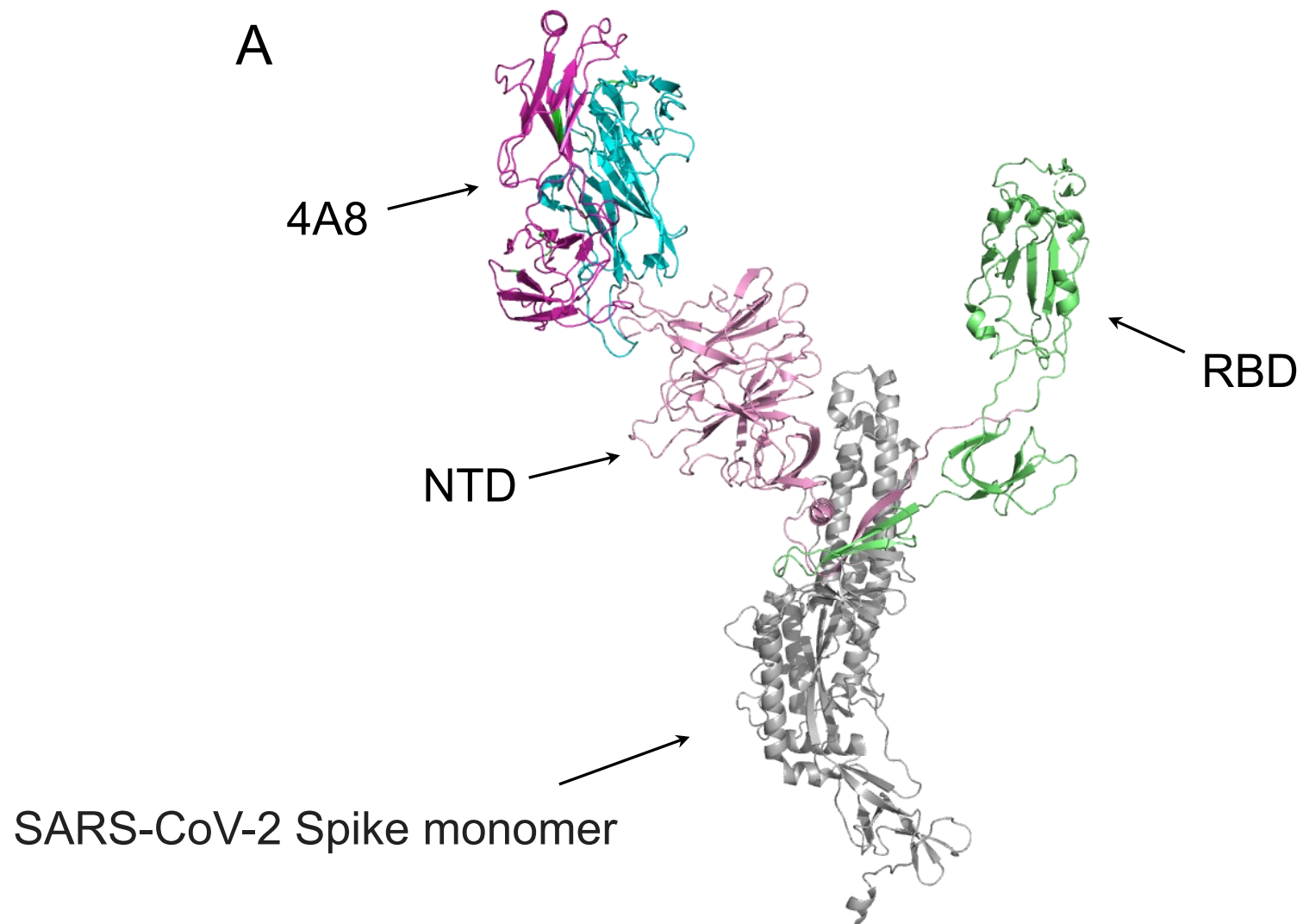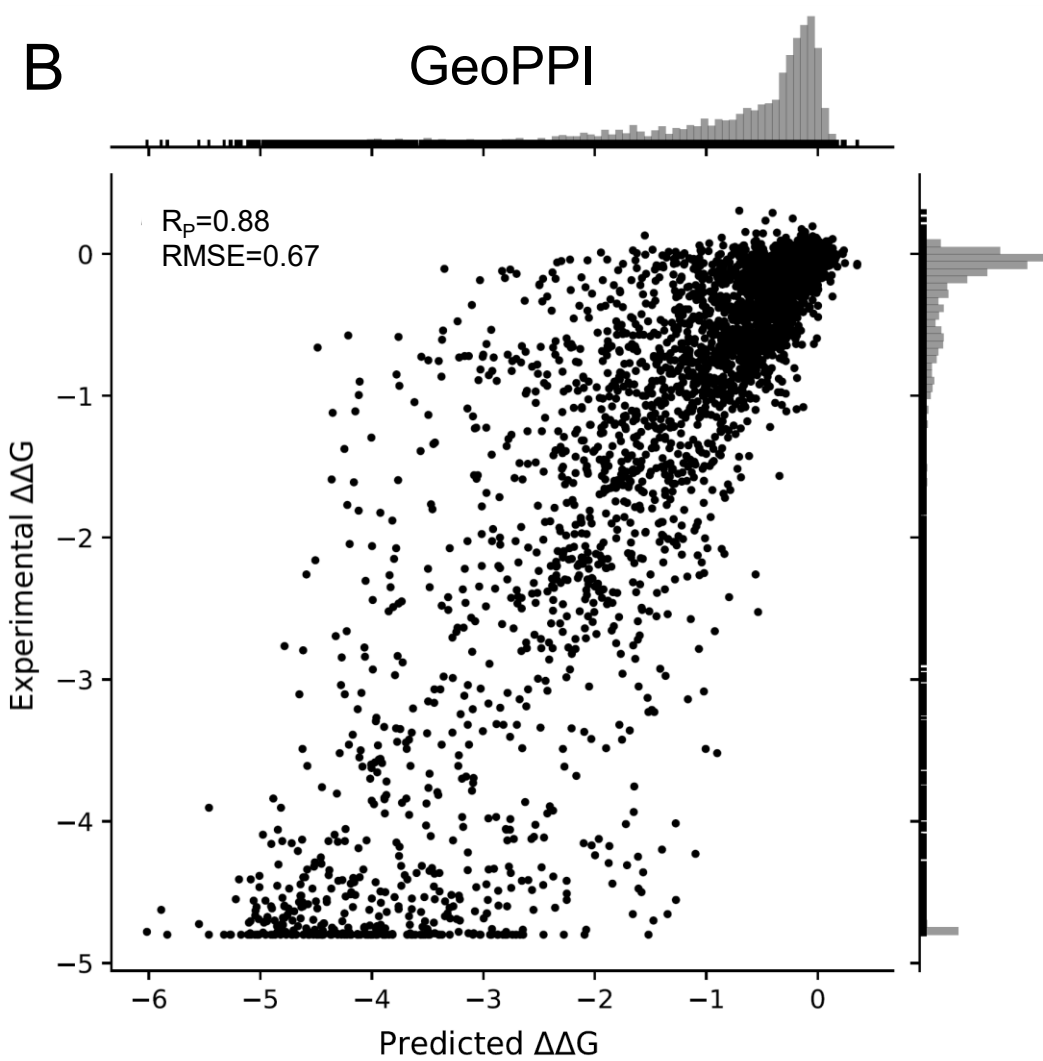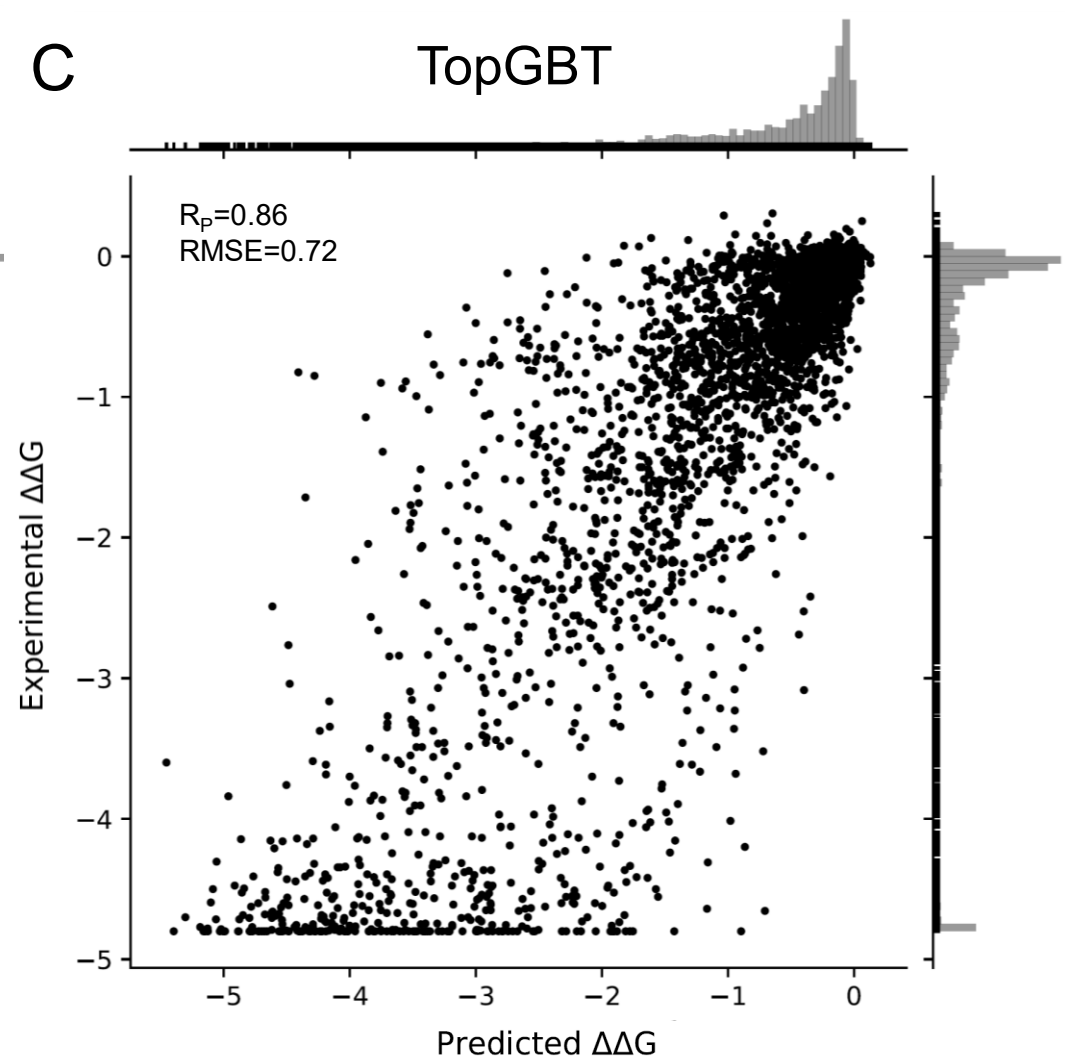

Supplement: S5 Fig — (A) The structure of the spike monomer of SARS-CoV-2 complexed with 4A8 (from PDB ID: 7c2l). (B) The performance of GeoPPI in the S3647 dataset in terms of the ten-fold CV test. (C) The performance of TopGBT in the S3647 dataset in terms of the ten-fold CV test. In the S3647 dataset, the binding affinity (ΔG) is measured by the apparent dissociation constant log10(KD,app). The Pearson correlation coefficient and root mean square error for each method are shown in the upper left corner of the subfigure. (PDF) [file pcbi.1009284.s007.pdf]

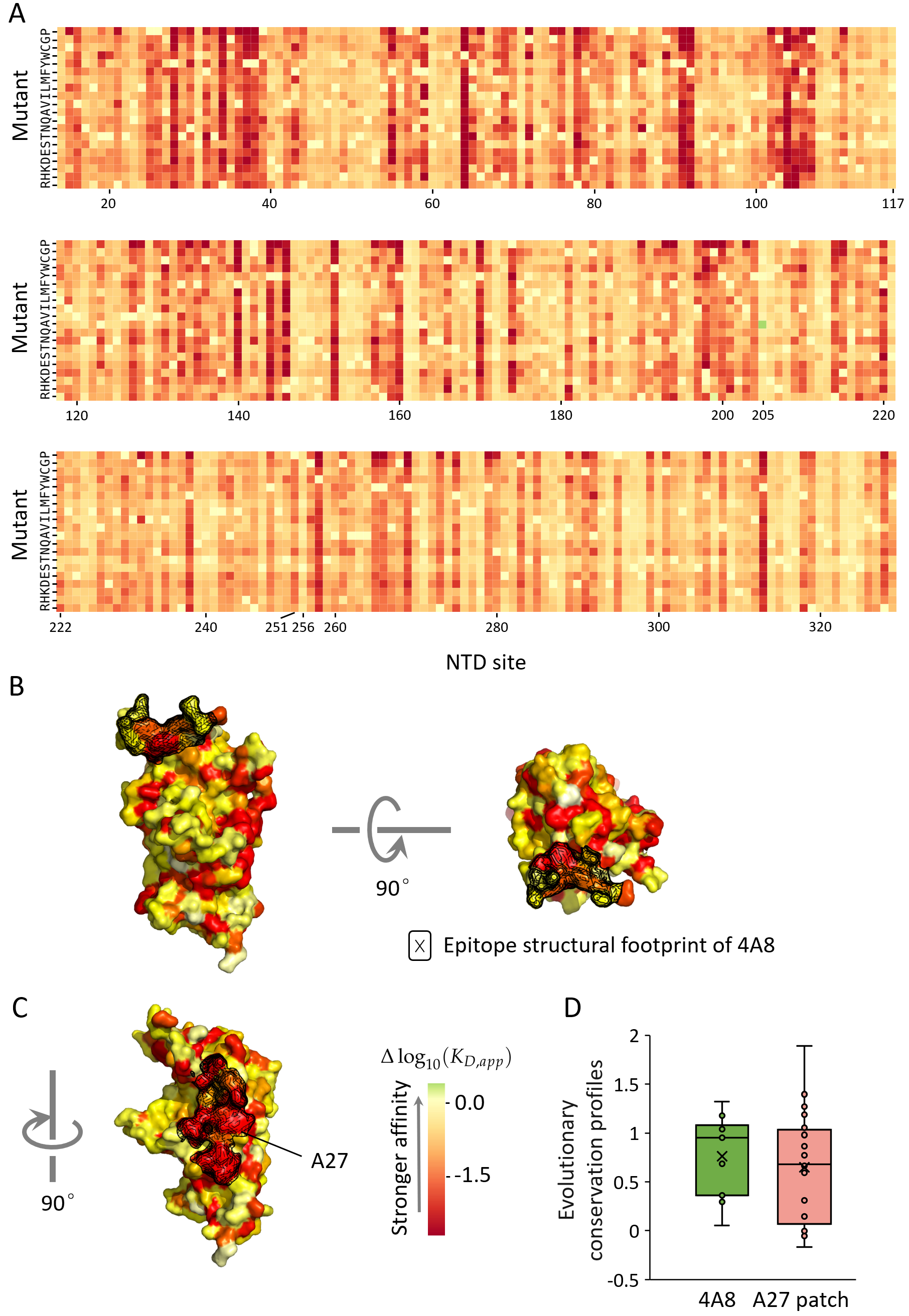

Supplement: S6 Fig — (A) Heatmaps representing how single-point mutations on the SARS-CoV-2 NTD impact the binding affinity with ACE2. The mutation that leads to an increase of binding affinity was circled in the green color. (B) The mutational constraint of the epitope of 4A8, an antibody targeting the SARS-CoV-2 NTD. The surface of NTD is colored according to the average mutational effects on the binding affinity with ACE2. (C) Identification of a patch of mutational constraint surrounding NTD residue A27. (D) The comparison of evolutionary conservation between the epitope of 4A8 and the newly identified A27 patch. The evolutionary conservation profiles of residues were calculated by ConSurf Database [74] based on the sequence alignment among 37 SARS-CoV-2 related sarbecoviruses summarized in Starr et al. [73]. (TIF) [file pcbi.1009284.s008.tif]
