# Supplementary material for: Deep geometric representations for modeling effects of mutations on protein-protein binding affinity
Source: PLoS Comput Biol. 2021 Aug 4;17(8):e1009284. doi: 10.1371/journal.pcbi.1009284 (PMC8366979; doi:10.1371/journal.pcbi.1009284)
Supplement: S1 Table — (PDF) [file pcbi.1009284.s009.pdf]

Features	Encoding method	Dimension
Atom type (C,N,O,S)	One-hot encoding	4
amino acid type	One-hot encoding	20
Is in the mutated chain	Binary values	1
Chain index	Sine and cosine functions	2
Is on the interface	Binary values	1
Coordinate (x, y, z)	Numeric values	3
