# Supplementary material for: Deep geometric representations for modeling effects of mutations on protein-protein binding affinity
Source: PLoS Comput Biol. 2021 Aug 4;17(8):e1009284. doi: 10.1371/journal.pcbi.1009284 (PMC8366979; doi:10.1371/journal.pcbi.1009284)
Supplement: S2 Table — (PDF) [file pcbi.1009284.s010.pdf]

Dataset	Description
S645	Single mutations, compiled from the AB-Bind dataset
S1131	Single mutations, compiled from the SKEMPI dataset
S1748	Single mutations, compiled from the SKEMPI 2.0 dataset
S641	Single mutations, mutations included in S1748 but not in S1131
S4169	Single mutations, compiled from the SKEMPI 2.0 dataset
S4191	Single mutations, compiled from the SKEMPI 2.0 dataset
S8338	Single mutations, S4169 plus all reverse mutations
M1101	Single and multiple mutations, compiled from the AB-Bind dataset
M1707	Multiple mutations, compiled from the SKEMPI 2.0 dataset
