# Supplementary material for: Deep geometric representations for modeling effects of mutations on protein-protein binding affinity
Source: PLoS Comput Biol. 2021 Aug 4;17(8):e1009284. doi: 10.1371/journal.pcbi.1009284 (PMC8366979; doi:10.1371/journal.pcbi.1009284)
Supplement: S4 Table — *: Results are obtained based on the released source code. (PDF) [file pcbi.1009284.s012.pdf]

Methods	S645	S1131	S4169	S4191	S8338
GeoPPI	**0.65**	**0.85**	**0.78**	**0.83**	**0.85**
TopGBT* [31]	0.56	0.78	0.76	0.79	0.82
TopNetTree* [31]	0.55	0.76	0.76	0.77	0.81
mCSM-PPI2 [60]	-	-	0.76	-	0.82
MutaBind2 [15]	-	-	0.74	0.79	0.81
BindProfX [30]	-	0.74	-	-	
Profile-score + FoldX [32]	-	0.74	-	-	
Profile-score [32]	-	0.68	-	-	
SAAMBE [33]	-	0.62	-		
mCSM-AB [49]	0.53	-	-	-	
TopCNN [31]	0.53	-	-	-	
FoldX [8]	0.30	0.46	0.27	0.40	0.44
BeAtMuSic[38]	0.28	0.27	-	-	
Dcomplex [29]	-	0.06	-	-	
