# Supplementary material for: Deep geometric representations for modeling effects of mutations on protein-protein binding affinity
Source: PLoS Comput Biol. 2021 Aug 4;17(8):e1009284. doi: 10.1371/journal.pcbi.1009284 (PMC8366979; doi:10.1371/journal.pcbi.1009284)
Supplement: S5 Table — The performance of GeoPPI and MutaBind2 on the M1101 dataset was obtained by the ten-fold CV. To have a fair comparison with the MutaBind2 on M1707, GeoPPI and FoldX were evaluated with the two-fold cross validation test. (PDF) [file pcbi.1009284.s013.pdf]

Methods	M1101	M707
GeoPPI	**0.78**	**0.89**
MutaBind2 [15]	-	0.87
Discovery Studio [13]	0.45	-
FoldX [8]	0.34	0.49
STATIUM [32]	0.32	-
mCSM-PPI [60]	0.31	-
DFIRE [28]	0.31	-
bASA [34]	0.22	-
dDFIRE [27]	0.19	-
Rosetta [35]	0.16	-
