# Supplementary material for: Deep geometric representations for modeling effects of mutations on protein-protein binding affinity
Source: PLoS Comput Biol. 2021 Aug 4;17(8):e1009284. doi: 10.1371/journal.pcbi.1009284 (PMC8366979; doi:10.1371/journal.pcbi.1009284)
Supplement: S6 Table — (PDF) [file pcbi.1009284.s014.pdf]

Complex	Mutation	experimental $\Delta\Delta G$	TopGBT	GeoPPI
1AK4	D:I419V	-0.17	2.01	0.48
1N8Z	L:T94S	0.31	1.07	0.47
1N8Z	L:Y92F	-0.21	0.42	0.47
1MHP	H:F99Y	0.76	2.56	2.67
1MHP	L:S91T	2.32	1.07	2.79
1MHP	L:S52T	8	1.06	3.72
1VDF	B:Y101F	2.03	3.29	0.70
1VFB	H:D58E	0.08	0.10	-0.30
1VFB	H:Y101F	1.6	2.88	0.22
2JEL	P:D69E	0.96	0.26	0.63
2JEL	P:E5D	0.41	0.59	0.24
2JEL	P:S64T	4.13	1.37	0.73
3FHM	L:Y50F	2.36	3.70	1.07
3FHM	L:Y96F	1.4	1.87	0.01
HM_1YY9	L:T97S	-0.7	-0.37	0.50
R_P	-	-	0.21	0.66
RMSE	-	-	2.19	1.71
