# Supplementary material for: Deep geometric representations for modeling effects of mutations on protein-protein binding affinity
Source: PLoS Comput Biol. 2021 Aug 4;17(8):e1009284. doi: 10.1371/journal.pcbi.1009284 (PMC8366979; doi:10.1371/journal.pcbi.1009284)
Supplement: S7 Table — The analysis was conducted on the S4169 dataset. (PDF) [file pcbi.1009284.s015.pdf]

Fold number	R_p	RMSE
2	0.512	1.493
3	0.514	1.490
4	0.515	1.489
5	0.517	1.488
6	0.517	1.488
7	0.518	1.486
8	0.519	1.485
9	0.520	1.485
10	0.522	1.483
