# Supplementary material for: Deep geometric representations for modeling effects of mutations on protein-protein binding affinity
Source: PLoS Comput Biol. 2021 Aug 4;17(8):e1009284. doi: 10.1371/journal.pcbi.1009284 (PMC8366979; doi:10.1371/journal.pcbi.1009284)
Supplement: S8 Table — The measurement was conducted using 1000 single-point mutations from a complex with 350 residues. Molecular dynamics with FoldX (MD-FoldX) and coarse-grained-umbrella sampling simulations (CG-US) are two molecular modeling methods for estimating the affinity changes upon mutations [75]. The computational time of TopGBT is obtained by running its source code. The test was conducted in the single CPU (Intel Core i7–4790K) or single GPU (NVIDIA GeForce GTX TITIAN X GPU) setting. †: Results were quoted from Patel et al. [75]. ‡: Results were quoted from Zhang et al. [15]. (PDF) [file pcbi.1009284.s016.pdf]

Methods	Time consumption using CPU (s)	Time consumption using GPU (s)
MD-FoldX[†]	$\sim 3.4 \times 10^6$	-
CG-US[†]	$\sim 7.6 \times 10^5$	-
FoldX[†]	$\sim 5.8 \times 10^2$	-
TopGBT	1.0×10^2	-
MutaBind2[‡]	43.2	-
GeoPPI	17.2	14.9
