# Supplementary material for: Deep geometric representations for modeling effects of mutations on protein-protein binding affinity
Source: PLoS Comput Biol. 2021 Aug 4;17(8):e1009284. doi: 10.1371/journal.pcbi.1009284 (PMC8366979; doi:10.1371/journal.pcbi.1009284)
Supplement: S11 Table — The hyperparameters in the geometric encoder include the hidden size Dg, the number of attention heads K, number of hidden layers L. We applied a coarse grid search approach over Dg ∈ {128, 256, 512}, K ∈ {2, 4, 6, 8, 16}, L ∈ {1, 2, 3, 4, 5} on the development set of the self-supervised learning dataset to select the best settings of these hyperparameters. (PDF) [file pcbi.1009284.s019.pdf]

Hyperparameter	Selected values
D_g	64
K	8
L	4
