# Supplementary material for: Deep geometric representations for modeling effects of mutations on protein-protein binding affinity
Source: PLoS Comput Biol. 2021 Aug 4;17(8):e1009284. doi: 10.1371/journal.pcbi.1009284 (PMC8366979; doi:10.1371/journal.pcbi.1009284)
Supplement: S1 Algorithm — (PDF) [file pcbi.1009284.s020.pdf]

Input: Dataset containing data points, the corresponding clusters and the fold size N

```
Get cluster $c$ and the corresponding list of data points $L_c$ that belongs to cluster $c$
Compute the size of list for each cluster
$\hat{C}$ = Argsort(the clusters $c$) in terms of the size of the corresponding clustered data points (the descending order)
folds = dict()
AvgN = dataset size/$N$
usedcluster = []
foldid = 0
for $i \in \hat{C}$ do
  if len(folds)==$N$: break, else: folds[foldid] = $L_i$
  threshold = AvgN+$\epsilon$, $\epsilon \in \mathcal{N}(0, 10)$
  for $j \in \hat{C}$ do
    if j in usedcluster: continue
    if $L_j \geq L_i$ : continue
    if len(folds[foldid])+len($L_j$)<threshold:
        folds[foldid] += $L_j$; usedcluster.append(j)
    else: foldid+=1
  end for
end for
for $j \in \hat{C}$ do
  if j not in usedcluster: folds[-1].append(j)
end for
return folds
```
